# Covalently Doped and Highly Oriented Covalent Organic Framework Thin Films

**DOI:** 10.1021/acsnano.5c03065

**Published:** 2025-07-10

**Authors:** Dayanni D. Bhagwandin, Brian M. Everhart, Kirt A. Page, Michael A. Altvater, Yao Yao, Rahul Rao, Kara Martin, Krishnamurthy Mahalingam, Cheri Hampton, Jonathan Ludwick, Griffin Roberts, Drake Austin, Md Sherajul Islam, Ly Tran, Arthur R. Woll, Lawrence F. Drummy, Ajit K. Roy, Tobin J. Marks, Antonio Facchetti, Tyson Back, Hilmar Koerner, Luke A. Baldwin, Nicholas R. Glavin

**Affiliations:** † Materials and Manufacturing Directorate, 33319 Air Force Research Laboratory, WPAFB, Ohio 45433, United States; ‡ BlueHalo, Dayton, Ohio 45432, United States; § National Research Council Research Associate, Air Force Research Laboratory, Wright Patterson AFB, Dayton, Ohio 45433, United States; ∥ Cornell High Energy Synchrotron Source, 5922 Cornell University, Ithaca, New York, New York 14853, United States; ⊥ Department of Chemistry and the Materials Research Center, 3270 Northwestern University, Sheridan Road, Evanston, Illinois 60208, United States; # Aerospace Systems Directorate, Air Force Research Laboratory, WPAFB, Dayton, Ohio 45433, United States; ¶ University of Dayton Research Institute, 300 College Park, Dayton, Ohio 45469, United States; ∇ School of Materials Science and Engineering, Georgia Institute of Technology, Atlanta, Georgia 30332, United States

**Keywords:** covalent organic framework, thin films, nanostructures, band structure engineering, semiconductors, sulfur heterocycles

## Abstract

Strict control of both crystallographic orientation and band structure is crucial in realizing future high performing semiconducting microelectronic devices based on 2D covalent organic frameworks (COFs). Due to the insoluble nature from extensive aromaticity, processing of these materials into well-ordered, highly crystalline thin films presents a great challenge. In this work, a strategy to enable controlled covalent doping of imine COF thin films with thiophene linkers is presented. By incorporating different aldehyde ratios of terephthalaldehyde (PDA) and 2,5-thiophenedicarboxaldehyde (TDA) with 1,3,5-tris­(4-aminophenyl)­benzene (TAPB) in a liquid–solid synthesis approach, a series of highly crystalline and uniformly oriented TAPB-PDA-TDA COF thin films with varying percentages of TDA linkers incorporated into the framework were obtained. In this case, incorporation of thiophene linkers up to 20% resulted in minimal disruption of the long-range crystallographic ordering. Moreover, a small amount of thiophene molecules covalently doped into the highly ordered structure results in a small reduction in the band gap and a corresponding increase in the work function and decrease in the valence band maximum, effectively behaving like a p-type dopant in conventional semiconductors. The covalently doped thiophene unit is shown to increase the π-conjugation through enhanced crystallinity in the framework, improving the electron delocalization in the structure.

## Graphical abstract

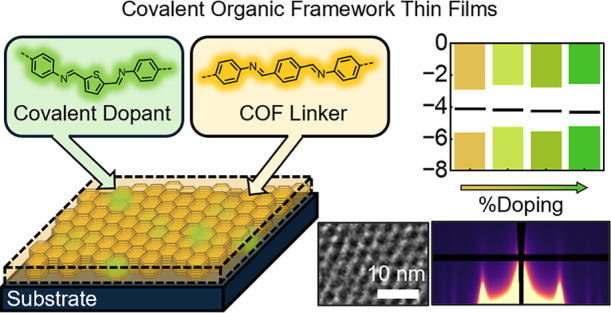

Band structure engineering is a crucial tool in modern microelectronics used for tailoring the electronic and optoelectronic properties of semiconductors. Alloying, doping, and heterostructure formation have all played a key role in controlling the band structure and design of new, inorganic semiconductor materials and devices at the forefront of modern, commercial electronics and optoelectronics.^1–3^ Similarly, developing methods to tune the band structure properties (in particular, the band gap) of organic semiconductors opens a new avenue to tailor material properties for the development of devices such as organic solar cells, organic light emitting diodes (OLEDs), and organic field effect transistors (OFETs).^4–6^ Of the multitude of emerging organic semiconductor materials, two-dimensional (2-D) COFs have shown considerable promise for future microelectronics applications as these materials have demonstrated mobilities as high as 165 cm^2^/(V s),^7^ ambipolar charge transport,^8,9^ tunable pore structure and chemistry for selective electronic sensors,^10^ and potential as channel materials in organic electrochemical transistors.^11^ Despite this potential, demonstration of band structure engineering through controlled doping or alloying in 2D COF thin films has yet to be achieved.

While COFs have demonstrated utility in a wide variety of electronic devices, limitations in synthesis and processing make obtaining thin films with a high degree of crystallinity and uniform orientation a great challenge.^12^ Limited solubility of monomers and reaction intermediates leads to aggregation and precipitation of material from solution while rapid nucleation times lead to the growth of polycrystalline COF films;^13^ especially in the case of 2D COFs.^12^ Furthermore, while computational methods can predict changes in the band structure as a result of structural modifications in the 2D COF lattice,^14–16^ experimentally determining the resultant electronic and optical properties has been limited to COF powders.^17–20^ Given that transport physics is affected by the crystallinity of a conjugated polymer^21,22^ and crystallite packing within semiconducting films,^23–25^ highly oriented COF thin films are desired to fully optimize performance and elucidate the effects of anisotropic transport^26^ associated with these uniquely layered 2D structures.

Recent work has demonstrated a solid–liquid growth technique that yields highly crystalline COF thin films with uniform orientation where layers are aligned parallel to the surface of the substrate and pores oriented perpendicular to the substrate.^11^ Covalent or electronic doping of these films is expected to be exceptionally influential on the electronic and optoelectronic properties, especially given the high degree of crystallinity and orientation throughout.^4^ Doping of COF powders with iodine or metal atoms have shown to modify properties of several COF species including WBDT COF,^27^ TF-TA COF,^28^ and other porphyrin COF structures.^29^ Hamzehpoor et al. recently described a covalent doping technique where a series of COF powders with varying levels of halogen substitution throughout were characterized.^30^ This type of “covalent” doping is exceptionally facile and fundamentally different from the classical doping approach of organic materials where dopants usually interact through noncovalent interactions.^31–38^ Instead, this approach is similar to doping in inorganic materials where heteroatoms exist in a single phase and are directly incorporated into the 2D lattice. For clarity, this work refers to the incorporation of TDA linkers into the COF structure as “covalent doping” similar to Hamzehpoor et al.,^30^ indicating the extent of covalently bound substituted linker molecules in the framework.

For this work, monomer units terephthalaldehyde (PDA) and 1,3,5-tri­(4-aminophenyl)­benzene (TAPB) are combined to form the imine-linked TAPB-PDA COF. Structural dopant, 2,5-thiophenedicarboxaldehyde (TDA) was chosen due to its electron-rich character, promotion of charge transfer efficiency,^39–41^ and similarity in structure and number of pi electrons to the complementary linker terephthalaldehyde (PDA). Films containing up to 20% TDA linker in the TAPB-PDA lattice show no disruption of long-range crystallographic ordering and provide evidence of band structure engineering with thiophene linker incorporation.

## Results and Discussion

### Doped COF Thin Film Synthesis

In this study, a series of crystalline, highly oriented, and covalently doped COF thin films are synthesized, using a previously published direct growth method,^11^ and then characterized for modulation in the optical and electronic properties. This thin film synthesis uses an interfacial setup, where acetic acid diffuses from the top aqueous layer into the bottom organic layer to controllably catalyze the reaction between organic monomers (shown in Figure 1a). After 24 h at room temperature, a COF thin film is formed on a Si/SiO_2_ substrate. Previous optimization of this method showed that acid concentration of the aqueous layer was related to the rate of particle formation in the organic layer and 1 M acetic acid formed films that were thin and smooth.^11^ In this work, doped films were synthesized using the condensation reaction of TAPB (1.0 equiv) with varying ratios of PDA and TDA (total aldehyde: 1.5 equiv).

**Figure 1:**
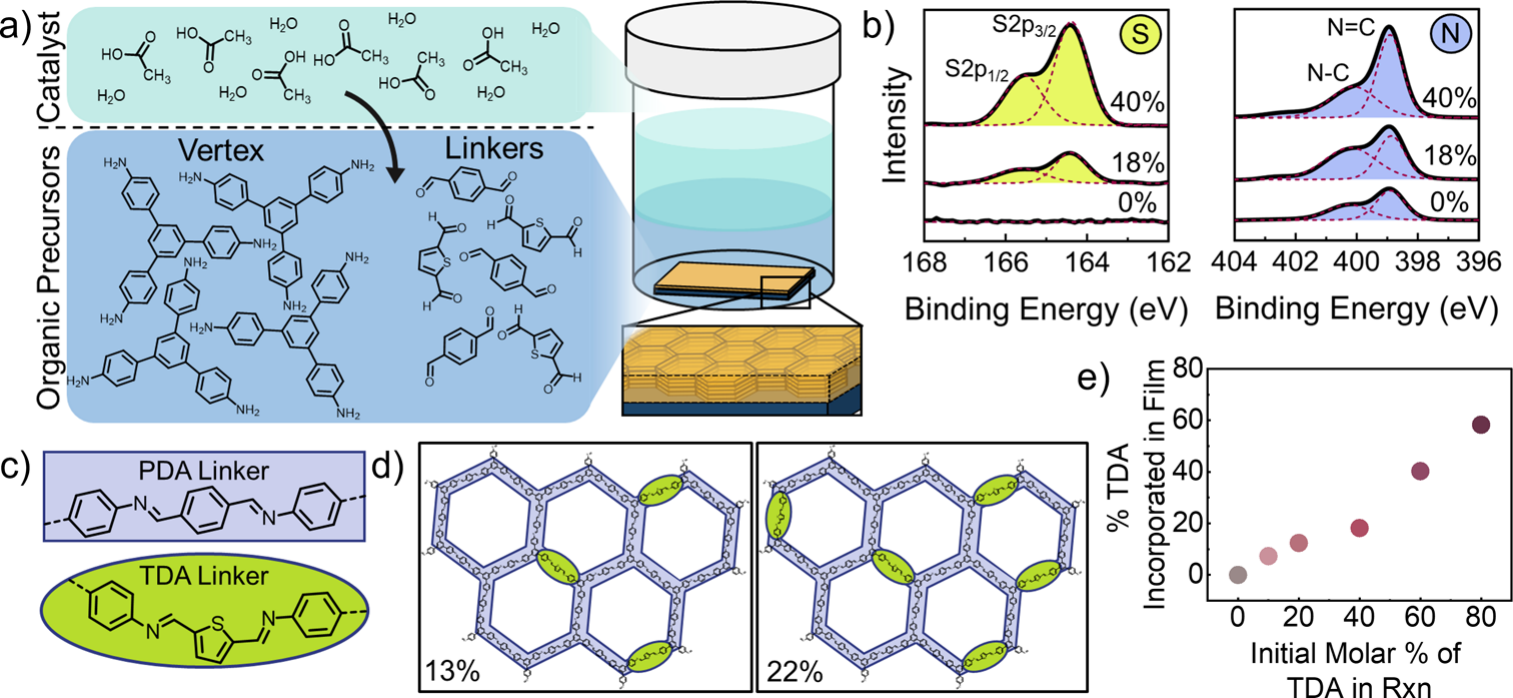
Covalently doped covalent organic framework thin film synthesis and characterization. (a) Diagram of COF thin film synthesis using an interfacial setup and parallel orientation obtained in COF thin film (bottom right). (b) Hi-res XPS scan of sulfur region from films containing 0%, 18%, and 40% TDA linker (left) and Hi-res XPS scan of nitrogen region from films containing 0%, 18%, and 40% TDA linker (right); deconvoluted peak fits shown as dashed red line. (c) Chemical structure of PDA linker (purple) and TDA linker (green) incorporation. (d) Structure of COF with 13% (left) and 22% (right) TDA linker incorporation. (e) Graph of TDA monomer (%) added versus TDA linker incorporated (%). *X*-axis shows the molar ratio of aldehyde monomers added and *y*-axis shows the ratio of sulfur to nitrogen calculated from XPS and represents the percent of TDA linkers in COF.

Aldehyde monomers were dissolved in methylene chloride and different volumes of each solution were used across the series to vary the overall TDA concentration (TDA: 0.98 mg/mL, PDA: 0.94 mg/mL, see Table S1 in the Supporting Information for more details). All reagents were added to a 20 mL scintillation vial, then a Si/SiO_2_ substrate approximately 1 cm^2^ was placed at the bottom. Aqueous acetic acid was pipetted slowly onto the organic layer ensuring minimal solvent mixing. After 5 days, the films were removed and briefly sonicated to eliminate larger particulates. Prior to characterization, the films underwent postsynthetic treatments to enhance the crystallinity via a solvent annealing step and drive off any remaining solvents with a subsequent heating step. More information regarding this step can be found in the Supporting Information. The films appear uniform in thickness, and height measurements by atomic force microscopy (AFM) images revealed thicknesses <100 nm (further information can be found in Supporting Information in Figures S1 and S2).

X-ray photoelectron spectroscopy (XPS) was used to confirm the presence of TDA linkers and characterize the extent of covalent doping within the structure. XPS spectra of the doped COF thin films show the expected presence of O 1s, N 1s, C 1s, and S 2p peaks (further information can be found in Supporting Information Figure S3 and Table S2). High resolution spectra of the N 1s orbital reveal a bimodal peak as reported in previous imine COFs,^42^ and the S 2p spectra indicate an increasing presence of sulfur at higher TDA:PDA ratios in the reaction (Figure 1b). In the spectra, the red dashed lines show the deconvoluted peak fits, which are identified as the corresponding S 2p_1/2_, S 2p_3/2_, N–C, and N=C peaks. The extent of TDA linkers in the film can be estimated by comparing the sulfur and nitrogen ratios as in the idealized COF structure, every linker is connected to two imine nitrogens. Note that this is an estimate for thiophene linker incorporation, as some thiophene units may be incorporated at the edge of grain boundaries or as defects connected to only one nitrogen. Figure 1c shows the structural differences between PDA linker (purple) and TDA linker (green) incorporation. Assuming this ratio, a 13% TDA or 22% TDA incorporation would result in thiophene substitutions in three or five out of every twenty-three linkers, respectively (Figure 1d). Figure 1e shows the comparison between the molar percentage of TDA initially added to the reaction versus percent of TDA linkers incorporated into the COF film. Note that here the molar percent is determined from the total moles of aldehyde initially added. These data suggest that TDA is less likely to be incorporated into the film than PDA because the resulting films have a lower ratio of TDA linkers than the ratio of TDA that was added to the reaction. To further confirm that TDA was incorporated into the film’s crystalline regions, transmission electron microscopy (TEM) and energy-dispersive X-ray spectroscopy (XEDS) was performed. The results show that sulfur can be detected in the same regions where the crystalline COF is observed (further details in Supporting Information Figures S4 and S5).

As a comparison model for the doped COF thin films investigated in this study, analogous powder samples of doped COF and undoped TAPB-PDA COF were synthesized using standard solvothermal techniques for imine-based COFs.^43^ The doped COF powder was synthesized using equivalent starting concentrations of TDA and PDA aldehydes, and the crystallinity and chemical composition were characterized and compared to the undoped COF powder sample. Powder X-ray diffraction (PXRD) spectra of both samples show similar peaks around 2θ = 2.8° (further information can be found in Supporting Information Figure S7), and reveals a pore size around 3 nm which is expected for TAPB-PDA COF.^43^ Figure 2a shows the chemical structure of each sample revealed by solid-state ^13^C nuclear magnetic resonance (ss-NMR) spectroscopy. Significant differences can be noted between the two spectra (Figure 2b) indicating the presence of thiophene linkers connected to vertices through imine bonding in the doped sample (blue spectra). In the doped sample, carbon *a*, which is associated with the imine carbon, shifts upfield to 150 ppm due to the presence of the incorporated thiophene. Additionally, carbons *b* and c shift downfield to 146 and 132 ppm, respectively. Carbon *c* now also appears at 132 ppm and can now be differentiated from carbon d, unlike in the undoped sample where both carbons in the ortho position appear together at 127 ppm. These results show that TDA is incorporated into the COF structure via covalent doping without disrupting the overall crystallinity of the powder sample. Additional analysis with Fourier-Transform Infrared (FT-IR) spectroscopy and Raman spectroscopy show the presence of unique COF peaks and the absence of starting materials (further information can be found in Supporting Information Figures S8–S10). FT-IR spectra confirm COF formation via the disappearance of amine and aldehyde peaks and the appearance of imine peaks in both doped and undoped samples; the doped COF further exhibits new or shifted peaks below 1500 cm^–1^ indicative of the incorporated thiophene linker, resulting in a distinct chemical structure. ^1^H NMR spectra from acid hydrolyzed doped COF powder reveals PDA and TDA starting monomers in almost equivalent concentrations confirming 50% TDA linker incorporation into the powder (see Supporting Information Figure S11 for more information).

**Figure 2:**
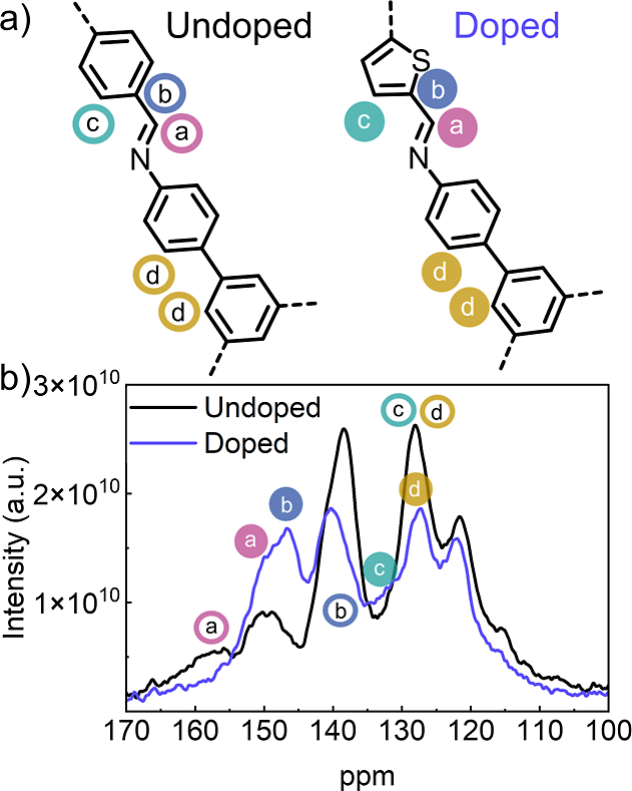
Unoped and doped COF powder analysis. (a) Structure of COF powders revealed by SS ^13^C NMR spectroscopy with colored circles depicting carbons with significant shifting between the analogous samples. (b) SS ^13^C NMR spectra of doped (blue) and undoped (black) COF powder samples with the corresponding shifted carbons.

### Crystallinity Analysis of Doped Films

To confirm the overall crystallinity of the COF thin films, grazing-incidence wide-angle X-ray scattering (GIWAXS) was performed on the COF samples. A representative set of 2D scattering images is shown in Figure 3a–c. The 1D scattering curves extracted from the GIWAXS images show strong peaks at 0.19, 0.34, 0.40 Å^–1^ in the undoped and 7% doped sample, consistent with peaks associated with TAPB-PDA COF.^43^ The peaks are associated with diffraction from the (100), (110) and (200) crystalline planes, respectively. These structural peaks can be seen in samples with TDA incorporation as high as 18% (Figure 3d), suggesting that TDA linkers can be incorporated into the lattice of the COF film without significantly disrupting crystallinity. The sharp, nearly perfectly vertical peak at 0.19 Å^–1^, associated with the (100) plane, indicates a high degree of orientation in the COF thin films with a defined pore structure of ca. 3 nm in diameter and 2D layering on the substrate. Moreover, the peaks also have a slight shift to higher *Q*_∥_ values in lower doped samples relative to the undoped samples, which is expected due to TDA’s smaller size relative to its analogous PDA linker. A previous report has shown that TAPB-TDA COF (also known as JUC-527) can be synthesized as a powder and exhibits a different pore shape with a size of 2.4 nm^44^ compared to TAPB-PDA COF with a reported hexagonal pore size around 3 nm.^43,45^

**Figure 3:**
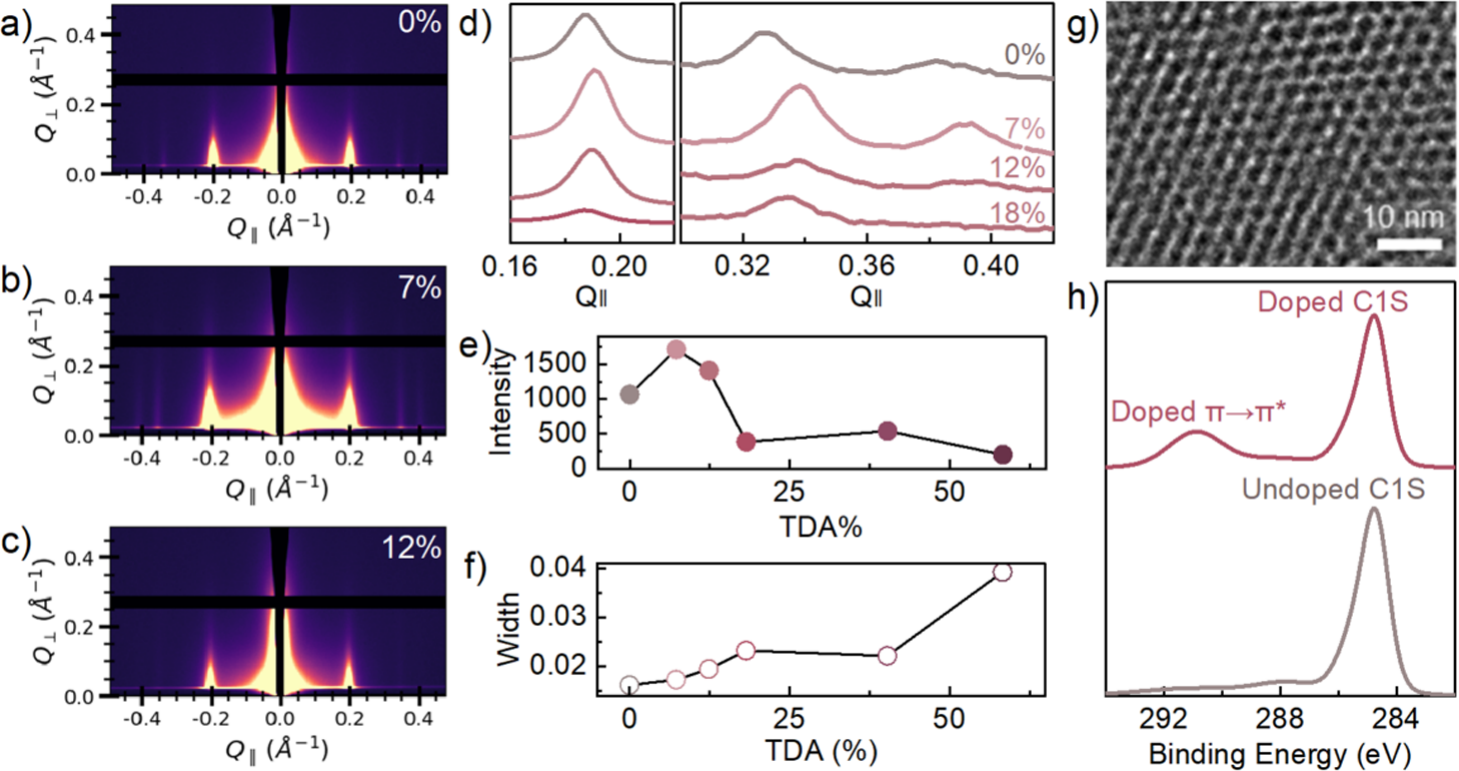
Crystallinity analysis of undoped and doped COF thin films. (a–c) Grazing-incidence wide-angle X-ray scattering images of 0%, 7%, and 12% TDA COF films showing peaks at ca. 0.2 Å^–1^ indicating crystallinity from the (100) plane. (d) 1-D line-cuts of 0%, 7%, 12%, and 18% TDA COF films showing peaks for the (100), (200), and (210) planes in low doped samples, respectively. (e) Plot of intensity of (100) peak across series of doped samples. (f) Plot of the full width at half-maximum of (100) peak across a series of doped samples. (g) Transmission electron microscope (TEM) image of doped COF synthesized with the addition of 50% TDA linker. (h) XPS of low doped COF (4% TDA) film showing π–π stacking in the C1S versus the undoped sample.

As shown in Figure 3e, COF thin films exhibit a precipitous loss in intensity of the (100) Bragg peak when doping exceeds approximately 12%. Under the assumption of identical GIWAXS measurement volume (sample thickness and beam dimensions, intensity and incidence angle), this decrease corresponds to a decrease in crystallinity with increasing covalent doping. This trend continues well beyond 12% doping, with measurable Bragg peak intensity disappearing entirely between 80% and 100% doping under the measurement conditions used (see Supporting Information Figure S12 for further details). Alongside this trend in the (100) Bragg peak intensity, its full width at half-maximum (FWHM) as a function of the percent of TDA linker present in the film is shown in Figure 3f and exhibits a similarly monotonic trend. The FWHM is an indicator of the grain-size of the crystalline regions, where a narrow peak corresponds to larger grain size and higher degree of order and broader peaks indicate less order. The intensity of the peaks is an indicator of the presence and quality of the crystallinity of the COF. As the level of doping increases there is a significant influence on the long-range crystallographic order. The fwhm of the peak increases with increasing doping, indicating a reduction in crystallite size at higher doping concentrations. Interestingly, low doped samples (<20% TDA) appear to have a higher scattering intensity than the undoped samples. This suggests that almost all pores will have at least one thiophene unit on the perimeter before the crystallinity of the thin film starts to be severely affected. Addition of the thiophene unit into the COF backbone may enhance crystallinity (to a certain extent) through increased noncovalent interactions between sulfur and nitrogen atoms within the layer,^46^ which would in turn improve the molecular planarity and packing. Furthermore, the crystalline domain orientation can be evaluated by examining the scattering intensity of the crystalline peaks as a function of the azimuthal angle, χ (see Figure S13). Typically for COFs, the azimuthal distribution of the scattering intensity can be used to quantify the degree of crystalline domain orientation using Herman’s orientation parameter (HOP).^47^ The COF Bragg peaks shown in the GIWAXS patterns in Figure 3a–c and the remaining films appear as vertical streaks (see Figure S12 Supporting Information for further details). The absence of curvature of these streaks indicates that the extent of the streaks arises from finite-domain size in the out of plane direction, and not from orientational disorder. The verticality of these streaks indicates a high degree of orientational order about the plane of the surface. Due to the lack of curvature, it would not be appropriate to quantify the degree of orientation using the HOP as it assumes a distribution of the scattering around the azimuth. We do not observe scattering along the azimuthal direction beyond the fwhm of the vertical peaks indicating a very high degree of orientational order of the domains for this COF series. Beyond establishing an upper bound of orientational disorder, a quantitative estimate of the degree of orientational disorder cannot be obtained from these patterns (preliminary analysis of this upper bound can be found in the Supporting Information in Figure S13). Nonetheless, the high degree of orientation observed from these COF samples indicates significant potential for specific transport properties.^48,49^

Additionally, Figure 3g shows a transmission electron microscopy (TEM) image of a high quality and highly oriented doped film synthesized using equivalent starting amounts of TDA and PDA. A porous crystalline structure is observed with a pore repeat distance of about 3 nm, consistent with PXRD and GIWAXS measurements. Additional TEM images, which highlight the consistency in crystallinity throughout for both the doped and undoped COF films, are also presented (see Supporting Information Figures S14–S20 for further details).

The long-term stability of these COF thin film samples was also investigated. Both doped and undoped COF samples were prepared, treated, and analyzed using GIWAXS to confirm their initial crystallinity. After one year in ambient conditions, the same set of samples underwent additional GIWAXS analysis. This analysis revealed that the crystallinity of these samples was retained, to an extent, even after one year. Furthermore, the crystallinity of these same samples showed a slight improvement after a second solvent annealing treatment. GIWAXS images illustrating the crystallinity of these samples can be found in Figures S21 and S22 of the Supporting Information. Additionally, a doped COF sample was synthesized and then processed, treated, and analyzed eight months later (see Supporting Information Figure S23), indicating that after eight months the sample retained strong crystallinity.

To further investigate the structural properties and attributes of the doped COF samples, the C 1s region of the XPS spectra was evaluated to determine evidence of high quality π–π stacking, as shown in Figure 3h. The π–π stacking peak at 291 eV is significantly enhanced in the doped samples and presents a very intense peak relative to other COF thin films.^50,51^ Enhanced π–π stacking could be attributed to the presence of thiophene units incorporated into the backbone, as previous literature has also shown improved stacking with the presence of additional thiophene units in conjugated polymer and COF systems.^52–54^

Additional characterization was carried out on the doped COF films using Raman spectroscopy. Figure 4a shows the spectra associated with all thin film samples across the series. Peaks associated with the starting materials are absent from all the film samples, indicating conversion of monomers to COF (see additional details in the Supporting Information Figure S10 and Table S3). Additionally, peaks associated with the imine C=N stretching modes at 1590 cm^–1^, aromatic ring chain vibrations at 1560 cm^–1^ and aromatic stretching modes for C–N (or C–C) at 1160 cm^–1^ were present in all the films.^55^ Higher doped samples show the appearance of a peak at 1460 cm^–1^ as well as the disappearance of the ring vibration peak at 1565 cm^–1^. The TAPB + TDA thin film sample with no PDA (100% TDA, top spectrum) shows an additional peak at 1365 cm^–1^. An additional peak at 1170 cm^–1^, when only the TDA dopant is present with no PDA, also appears. This peak can be attributed to the C–N/C–C stretching mode for the thiophene-linked imine groups. Further analysis through peak fitting of the lower doped samples (<20%) was conducted to divulge trends in peak intensity and frequency in relation to the concentration of TDA dopant. Figure 4b (black arrow and filled data points, left axis) shows the linear change in the ratio of Raman peak intensities of the 1170 cm^–1^ and 1165 cm^–1^ peaks. As more TDA is added the 1170 cm^–1^ peak increases in intensity while the peak at 1165 cm^–1^ decreases. The same trend is also seen in the ratio of peak intensity for the C=N stretching mode at 1590 cm^–1^ and the aromatic ring vibration 1565 cm^–1^ peaks (hollow arrow and data points, right axis). Furthermore, Figure 4c shows that the peak at 1590 cm^–1^ shifts to a lower frequency as the percentage of TDA is increased, presumably due to the electron donating character of the thiophene.^56,57^ This peak frequency red shift has also been observed before in other 2D materials like doped graphene oxide.^58^ These observed trends could be used as a characterization method to rapidly determine the amount of TDA linker incorporated in COF thin films, rather than performing other extensive characterization.

**Figure 4:**
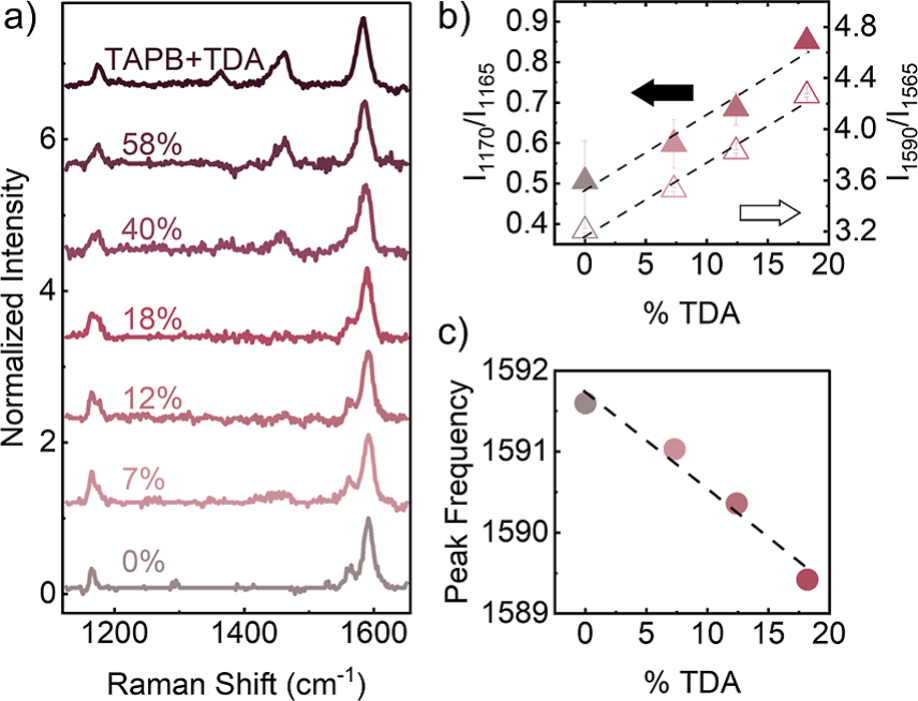
Raman analysis of undoped and doped COF films. (a) Raman spectra of series of undoped and doped COF films. (b) Graph of %TDA in COF film versus ratio of 1170 cm^–1^ and 1165 cm^–1^ peak intensities (solid labels/left axis) and ratio of 1590 cm^–1^ and 1565 cm^–1^ peak intensities (hollow labels/right axis). (c) Graph of %TDA in COF film versus 1590 cm^–1^ peak frequency.

### Density Functional Theory Calculations and Band Structure Analysis

To understand the impact of the covalently bound TDA dopant on the electronic transport properties and band structure, analysis of the density functional theory (DFT)-computed band structure and the electronic states nearest the Fermi level was performed (Figure 5). The model is built from the TAPB vertex and linker molecules (PDA/TDA) whose isolated electronic energy levels show partially π-conjugated electron densities and energetically isolated highest occupied molecular orbital (HOMO) and lowest unoccupied molecular orbital (LUMO) states (see Figure S24 in the Supporting Information). When placed into an ordered lattice, extended π-conjugated molecules exhibit both intra- and intermolecular electronic wave function overlap leading to the formation of broadly dispersing energy bands. The resulting in-plane band structure given in Figure 5c,d exhibits both flat and dispersing bands near the valence and conduction band edges. It is important to note that in Figure 5c,d, the *y*-axis depicts the energy with respect to the predicted Fermi energy (*E*_F,TH_). This is because in DFT calculations, the Fermi level is defined as the energy at which the occupancy probability of electronic states is 50% at absolute zero temperature. *E*_F,TH_ functions as a reference for the electronic structure and is derived from the electron distribution inside the computed density of states (DOS). This value is intrinsically linked to the idealized, periodic, and defect-free structure represented in DFT and is referenced in relation to the vacuum level or an internal energy zero, contingent upon the employed approach. The dispersions of the conduction and valence bands are controlled by the symmetries of the molecular lattice and are recognized to arise from the Kagome and honeycomb-trimer sublattices of the COF crystal, respectively (see Supporting Information Figure S22).^59^ As observed in both organic and inorganic crystals, these lattices exhibit flat, isolated bands which can realize the situation where the electronic kinetic energy becomes smaller than electronic interaction energies leading to correlated electronic ground states such as Mott insulators, generalized Wigner crystals, itinerant ferromagnetism, topologically nontrivial phases, and fractional quantum Hall states. The realization of these states in 2D COFs will depend on the degree of delocalization of π-electrons which can be improved through covalent doping by reducing the average energy difference between core and linker π orbitals.^60^ Thus, when combined with the ability to control the Fermi energy with respect to the valence and conduction bands (through techniques such as covalent doping), COFs may realize a highly tunable system to explore fundamental physics and tailor new functionalities for electronic and electrochemical devices.

**Figure 5:**
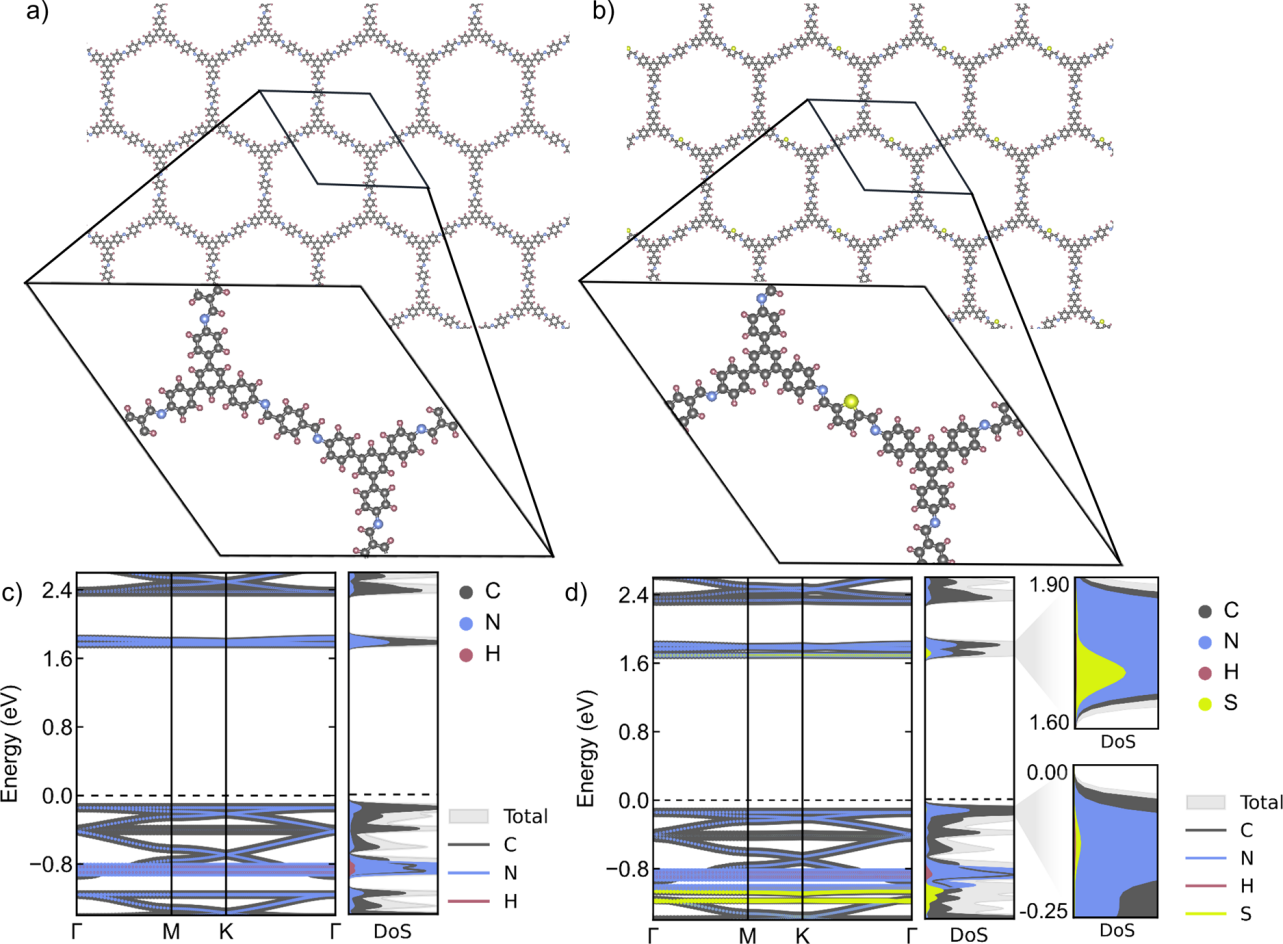
Density functional theory analysis. (a) Structure of undoped TAPB-PDA COF with the unit cell shown in inset. (b) Structure of doped COF (33% TDA) with the unit cell shown in inset. (c) Density of states for undoped TAPB-PDA COF. (d) Density of states for doped COF (33% TDA).

The introduction of covalently doped thiophene units into the TAPB-PDA COF lattice exhibits similar overall band structure to the undoped COF, as shown in Figure 5c,d, with specific atomic contributions to the energy bands and density of states (DoS) also indicated. Energy bands are plotted with respect to the Fermi energy versus momentum along a path through the indicated high symmetry points in the Brillouin zone (see further information in Supporting Information Figure S26). Most of the sulfur-like states are localized between −1.05 and −1.20 eV, however, states also appear at both the conduction and valence band edges. The flat conduction band contains TDA-like states and is shifted toward a lower energy, reducing the band gap slightly. Similarly, the flat valence band edge obtains a small degree of TDA-like character. The incorporation of the asymmetric TDA molecule removes the inversion symmetry of the lattice, breaking the degeneracy of the Dirac points through mixing of TDA and PDA states, opening a small gap at the Dirac points.

To experimentally determine the degree that covalent doping affects the electronic and optical properties of the COF film, ellipsometry and XPS were employed to construct an energy band diagram at different doping conditions. Figure 6a,b show the *n* (refractive index) and k (extinction coefficient) plots respectively for the covalently doped COF series at low doping concentrations. The 7% TDA-doped sample revealed the highest n value of all samples tested as well as the highest absorption in the near-infrared (NIR) region. A higher refractive index in is expected to be a result of the increased crystallinity and π-conjugation, which can increase the linear polarizability and thus the refractive index.^61^ Moreover, the broadening of the 700–1000 nm absorption in ellipsometry has also been attributed to extended π-conjugation, often as a result of doping. In either case, this increased charge delocalization is expected to be the result of in improved π–π stacking (as indicated in Figure 3h),^54,62^ enhanced molecular planarity from noncovalent interactions,^52–54,63^ the electron donating characteristic of the conjugated thiophene units,^56^ and the resulting robust crystallinity (as seen in Figure 3b)^7,64^ in the covalently doped TDA COFs. Further experimental and theoretical studies are necessary to determine the exact interplay between physical and chemical factors in these highly oriented doped COF films.

**Figure 6:**
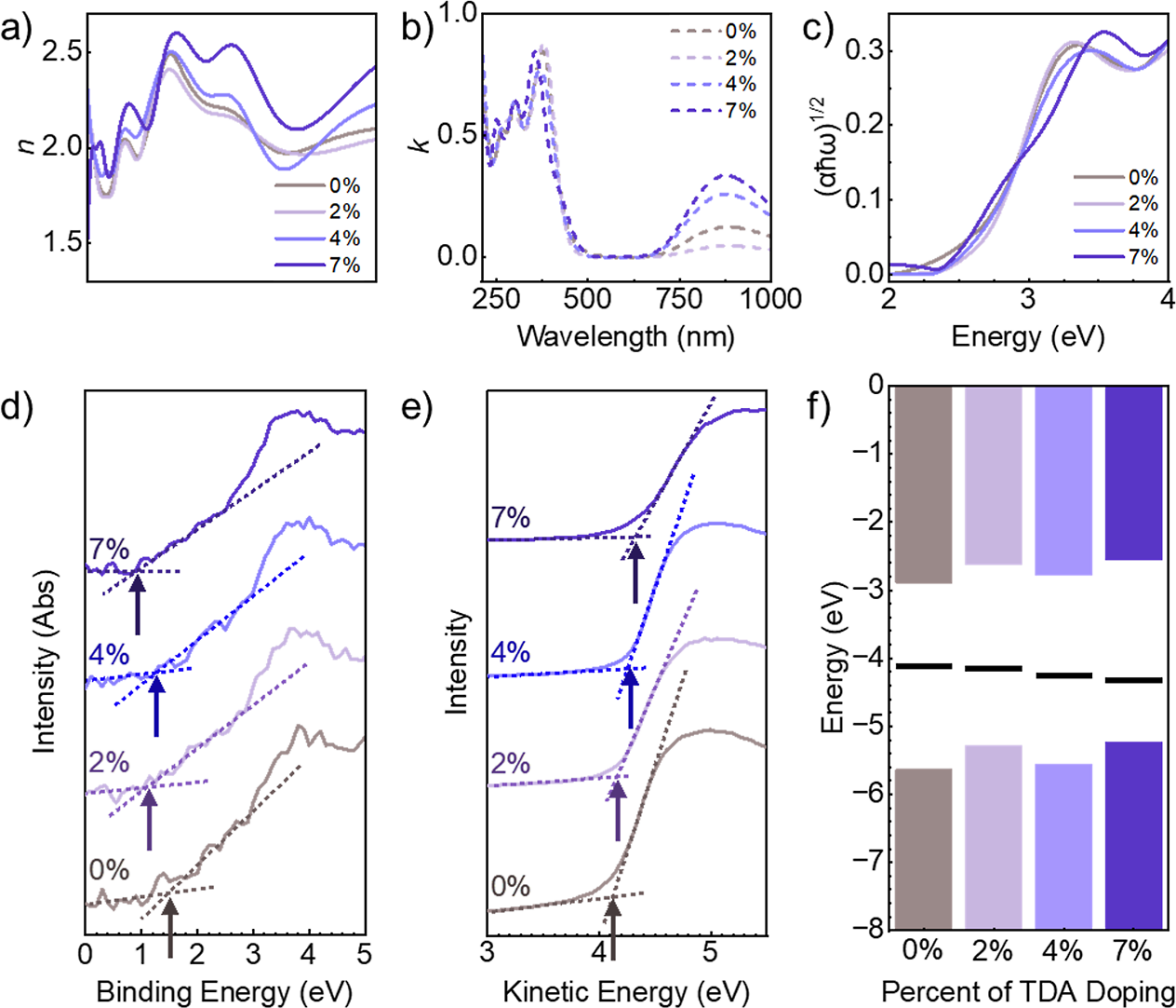
Electronic and optical properties of thiophene doped COF films. (a) *n* (refractive index) for doped series. (b) *k* (extinction coefficient) value for doped series. (c) Tauc plot for the evaluation of band gap of doped series. (d) Evaluation of VBM of the doped series. (e) Evaluation of work function of doped series. (f) Band diagram of doped series.

Figure 6c shows the resulting Tauc plots used to determine the band gap across the doped series. The band gaps are determined to be 2.74, 2.66, 2.79, and 2.68 eV for the 0%, 2%, 4%, and 7% doped samples, respectively. The valence band maximum (VBM) (Figure 6d) decreases from the original undoped value of 1.5 eV, with doped samples 2%, 4%, and 7% exhibiting a VBM of 1.1, 1.3, and 0.9 eV, respectively. Experimental measurements of the work function (WF) are depicted in Figure 6e and show a slight increase in binding energy over the doping concentration explored. The resulting work function values were determined to be 4.13, 4.18, 4.26, and 4.33 eV for the 0%, 2%, 4%, and 7% doped samples, respectively. This result is consistent with the electron-rich nature of thiophene versus benzene which shifts the energy of fill states to greater energies.^57^ This shift in VBM reveals a similar phenomenon as p-type doping, due to enhanced electron delocalization from interrupting the structure with thiophene units.^65^ Ellipsometry measurements indicate only a minimal change in bandgap due to increased covalent doping, as expected from the DFT results which indicate no new states within the bandgap and some new occupied states only at the valence and conduction band edges. Figure 6f shows the resulting energy band diagram indicating that the covalent doping of TAPB-PDA COF with TDA linkers results in a similar effect to p-type doping in conventional semiconductors, where the Fermi level moves closer to the valence band. In this case, however, as the thiophene unit does not have an acceptor unit available, this is presumably due to the delocalization of π-electrons across the molecular framework from improved crystallinity and π-conjugation. The experimentally measured Fermi energy differs slightly from the DFT-predicted Fermi level due to several factors. Namely, experimental Fermi levels may be affected by external variables such doping, defects, adsorbates, surface termination, and temperature, which are generally not considered in conventional DFT calculations. Furthermore, DFT, especially when employing prevalent exchange–correlation approximations such as Perdew–Burke–Ernzerhof (PBE), is recognized for its tendency to underestimate band gaps and may alter the relative locations of the valence and conduction bands.

Electronic and optoelectronic devices leveraging the chemical versatility and semiconducting performance of COFs will only be realized through improved crystallinity, orientation, and property control. Thus, when combined with the ability to control the Fermi energy with respect to the valence and conduction bands (through techniques such as covalent doping), COFs may realize a highly tunable system to explore fundamental physics and tailor new functionalities for electronic and electrochemical devices. To continue to expand the library of suitable COF films for microelectronic devices, it will be necessary to synthesize series of crystalline films that are doped to different capacities and with different monomers. Future work aims to incorporate dopants that can be used to enhance crystallite size as well as redox-active capabilities and hydrophilicity. Incorporating both redox active dopants and hydrophilic dopants offers potential for electron transport through oxidation or reduction,^66^ while maximizing interactions between aqueous electrolytes and the COF film^67^ in electrochemical devices such as organic electrochemical transistors (OECTs).

## Conclusions

Electronic and optoelectronic devices leveraging the chemical versatility and semiconducting performance of COFs require reliable approaches to band-structure engineering while preserving or improving crystallinity, orientation, and property control. In this study, covalent doping of thiophene linkers into a TAPB-PDA COF retained, and even improved, the crystal structure in thin films with upward of 20% doping. Several characterization techniques, including solid-state NMR spectroscopy, XPS, Raman spectroscopy, and TEM indicate TDA incorporation into the lattice structure of the framework. Experimental results and density functional theory (DFT) confirm band structure modification resulting in an increase in work function and decrease in VBM, akin to p-type doping in conventional semiconductors. This is due to the increase in charge transport capacity upon the partial incorporation of the covalently bound TDA dopant which results in enhanced π–π conjugation and increased crystallinity. In summary, this work provides a detailed investigation of covalently doped, highly ordered COF thin films, revealing the correlations between their composition, crystallinity, and electronic properties through comprehensive characterization.

## Methods

### Reagents

All reagents were purchased from commercial suppliers. 1,3,5-tris­(4-aminophenyl)­benzene (TAPB) was purchased from Ambeed, Inc. with a 97% purity. 1,4-terephthalaldehyde (PDA) was purchased from Aldrich with a 99% purity. 2,5-thiophenedicarboxaldehyde (TDA) was purchased from Sigma-Aldrich with a 99% purity.

### Data

All data were plotted using Origin Pro.

### Doped COF Thin Film Synthesis

A solution of 1,4-terephthalaldehyde (PDA) in methylene chloride (DCM) and a solution of 2,5-thiophenedicarboxaldehyde (TDA) in DCM were added to a 20 mL scintillation vial so that the total volume was 8 mL (see Table S1). Then 1,3,5-tris­(4-aminophenyl) benzene (TAPB) (13 mg) was added to the vial. Next, the vial was briefly sonicated. A Si/SiO_2_ substrate (approximately 1 cm^2^) was then placed at the bottom of the vial. Then 8 mL of aqueous acetic acid (1 M) was slowly pipetted on top of the organic layer. Crystalline film formation takes place in the first 24 h but these films were left undisturbed for 5 days. In the low doped sample series, the substrates were placed upside down in the vial, so that the COF film that that formed on the bottom would have better uniformity. Following the synthesis step, the films were subjected to an annealing step using a modified procedure.^11^ Films were saturated with a dioxane, mesitylene, and 10.9 M acetic acid (4.1:1:1), v/v)) solution. Then they were placed in a chamber above the same solvent and subjected to the vapors by heating at 70 °C for 1 h. Following this, they were placed on a hot plate and heated at 120 °C for 15 s.

### COF Powder Synthesis

As a comparison model, a covalently doped COF powder and undoped (TAPB-PDA) COF powder was also synthesized using traditional solvothermal methods.

### Doped COF Powder Synthesis

1,3,5-tris­(4-aminophenyl) benzene (TAPB) (150 mg, 0.43 mmol, 1.0 equiv), 1,4-terephthalaldehyde (PDA) (43 mg, 0.32 mmol, 0.75 equiv), and 2,5-thiophenedicarboxaldehyde (TDA) (45 mg, 0.32 mmol, 0.75 equiv) were added to a 48 mL glass pressure vial which was then briefly sonicated. To this mixture was added 17 mL of a dioxane, mesitylene, and 10.9 M acetic acid mixture (4.1:1:1). The reaction was heated to 70 °C for 3 days. After, the solid was removed and washed with methanol and acetone. Then it was gently dried and used for characterization.

### Undoped (TAPB-PDA COF) Powder Synthesis

1,3,5-tris­(4-aminophenyl) benzene (TAPB) (150 mg, 0.43 mmol, 1.0 equiv) and 1,4-terephthalaldehyde (PDA) (85 mg, 0.64 mmol, 1.5 equiv) were added to a 48 mL glass pressure vial which was then briefly sonicated. To this mixture was added 17 mL of a dioxane, mesitylene, and 10.9 M acetic acid mixture (4.1:1:1). The reaction was heated to 70 °C for 3 days. After, the solid was removed and washed with methanol and acetone. Then it was gently dried and used for characterization.

### Raman Spectroscopy

Raman spectra were collected using 514.5 nm excitation wavelength in a Renishaw inVia Raman microscope with an 1800 lines/mm grating. A 50× objective lens was used to focus the incident laser beam on the samples, which were irradiated by an effective laser power of ∼0.3 mW. Raman spectra were collected from the samples with 30 s acquisition times. One acquisition was collected from three different regions on a given film, and the median value was taken at each point.

### Atomic Force Microscopy

AFM images were collected using a Bruker Dimension Icon with Scan Asyst in noncontact tapping mode. The images were quantitatively analyzed for roughness and thickness using NanoScope Analysis.

### Transmission Electron Microscopy

Projection images were recorded on a 4K Ceta CCD camera. Prepared TEM grids were imaged by conventional bright-field TEM and high-angle annular scanning transmission electron microscopy (HAADF-STEM) using a Thermo Fisher Scientific Talos 200C and 200-FX TEM (ThermoFisher Scientific Inc.) operated at an acceleration voltage of 200 kV. Furthermore, elemental mapping was performed by X-ray energy dispersive spectroscopy (XEDS) under HAADF-STEM imaging conditions, wherein XEDS data acquisition and processing was performed using the ChemiSTEM (ThermoFisher Scientific Inc.) instrumentation and Esprit 1.9 (Bruker Inc.) software.

### Grazing-Incidence Wide-Angle X-ray Scattering (GIWAXS)

The grazing-incidence X-ray scattering measurements were carried out at the Functional Materials Beamline (FMB) of the Materials Solutions Network at the Cornell High Energy Synchrotron Source (MSN–CHESS). An X-ray beam energy of 9.7 keV (λ = 1.28 Å) was selected using the 111 reflection of a single-bounce, HPHT diamond monochromator.^68^ Harmonic rejection and vertical focusing are provided by a 1 m long, bendable, rhodium-coated monochromatic mirror located approximately 7 m upstream of the experimental hutch at an incident angle of 4 milliradians. Experiments were carried out in “bulk-beam” mode and the monochromatic mirror was used to focus the beam into a spot approximately 0.045 × 0.5 mm^2^ at the sample position, with a total flux of approximately 10^12^ photons/second^i^ at 100 mA beam current.^69^ The samples were mounted on a 4-axis goniometer and aligned using a downstream ion chamber. Experiments were performed over a range of incident angles, both below and above the film critical angle. Scattering images were collected on a Pilatus 300 K detector (Dectris, Baden, Switzerland) with a sample-to-detector distance of ca. 81.6 cm. Detector images were calibrated using silver behenate to convert the images to *q*-space. Software^70^ was used to analyze the scattering images and to produce intensity versus scattering vector, *q*(Å^–1^), plots.

### X-ray Photoelectron Microscopy (XPS)

X-ray photoelectron spectroscopy (XPS) measurements were performed using an Argus CU system (Scienta Omicron) equipped with a monochromatic Al Kα source (hν = 1486.7 eV) at 300W under a base pressure of 3 × 10^–10^ mbar. Survey spectra were acquired with a pass energy of 89.05 eV, a dwell time of 0.1 s, −1 eV step increments, and a 1.5 eV charge neutralizer setting, using a large aperture (∼1.93 mm spot). High-resolution elemental scans (S 2p, N 1s, and C 1s) were recorded at 20 eV pass energy, 0.05 eV step size, 0.1 s dwell time. The energy scale was calibrated by referencing the C 1s peak to 284.8 eV. Valence band maximum measurements were collected using a Mg Kα source (1253.6 eV), a pass energy of 20 eV, 0.05 eV step size, 0.1s dwell time. Work function measurements were performed by applying a negative bias to enhance secondary electron emission, using 5 acquisitions, a 0.01 eV step size, and a 2 eV pass energy. For lateral uniformity, a 3 × 3 grid of measurements was performed at each sample location with a 200 μm spot size. The work function was measured for each measurement, and an average value was calculated for each film.

### Density Functional Theory (DFT) Calculations

The periodic COF structure was simulated with Vienna Ab Initio Simulation Package (VASP) within the MedeA package, employing the Perdew–Burke–Ernzerhof (PBE) functional for exchange–correlation effects. A 3 × 3 × 1 Γ-centered *k*-point mesh was utilized for Brillouin zone sampling, and the wave functions were enlarged using a plane-wave basis set with a 450 eV kinetic energy cutoff. To reduce interactions between periodic pictures in the 2D thiophene linked COFs, a vacuum layer of 15 Å was included. The atomic structures were optimized until the forces on all atoms were below 0.02 eV/Å, with the energy convergence threshold established at 10^–4^ eV for self-consistent field iterations for optimizations and 10–5 eV for static computations.

### Powder X-ray Diffraction (PXRD)

Data was collected on a Rigaku Smartlab diffractometer with a Cu Kα (1.5406 Å) X-ray radiation source and was operated at 40 kV and 44 mA. The scan speed was 2.0°/min.

### Solid State NMR Spectroscopy

Solid-state NMR experiments were performed at 9.4T using a Bruker Avance III wide-bore NMR magnet equipped with a 3.2 mm MAS probe at room temperature. The samples were packed into zirconia rotors and capped with Vespel caps. Samples were studied using ^1^H–^13^C cross-polarization (CP), starting with a generic CP procedure from TopSpin followed by parameter optimization (POPT) for the COF samples. The optimized procedure used a dwell time (DW) of 10 μs. Recycle delay (D1) of 10 s 1H 90° and 180° pulses were 2.6 and 5.2 μs, respectively. 1H power level during decoupling (PLW12) was 75W and 1H power level during contact (SPW0) was 50W. Contact time on the ^13^C channel was 2000 μs with a power level (PLW1) of 100 W. Samples were spun at 8 kHz and 6000 scans were collected.

### Solution Phase NMR Spectroscopy

^1^H NMR spectra was obtained on Bruker 400 MHz spectrometer.

### Spectroscopic Ellipsometry

A J. A. Woollam RC2 ellipsometer was used to characterize the optical constants of the covalently doped COF films. Optical dispersion data were collected from 210 to 2500 nm at 50–60° angles with a step size of 5°. Optical dispersion data analysis was performed using CompleteEASE v6.55 (J.A. Woollam) with a B-Spline model used to determine the optical constants from the polarization change amplitude ratio and phase difference. Each model consisted of a silicon substrate with a 300 nm thick oxide layer along with the COF film. The fitting of the model yielded an estimated film thickness of 38–54 nm with a surface roughness of 7–9 nm.

## Supplementary Material



## Notes

1. Synthesis information 2. Supplementary Figures (photos of films, photos of powder, microscopy images of films, survey XPS scans, sulfur mapping, PXRD, FT-IR spectra, Raman spectra, ^1^H NMR spectra, GIWAXS, azimuthal scattering, HOP calculation, HOMO and LUMO states) (PDF)

## Abbreviations

DCM: dichloromethane
GIWAXS: grazing-incidence wide-angle X-ray scattering
HOMO: highest occupied molecular orbital
LUMO: lowest unoccupied molecular orbital
OECT: organic electrochemical transistor
COF: covalent organic framework
PDA: terephthalaldehyde
TDA: thiophenedicarboxaldehyde
TAPB: 1,3,5-tris­(4-aminophenyl)­benzene
OLED: organic light emitting diodes
OFET: organic field effect transistor
2-D: two-dimensional
AFM: atomic force microscopy
XPS: X-ray photoelectron spectroscopy
TEM: transmission electron microscopy
XEDS: energy-dispersive X-ray spectroscopy
PXRD: powder X-ray diffraction
NMR: nuclear magnetic resonance
FT-IR: Fourier-transform infrared
HOP: Herman’s orientation parameter
DFT: density functional theory
DoS: density of states
*n*: refractive index
*k*: extinction coefficient
NIR: near-infrared
WF: work function
KE: kinetic energy
VBM: valence band maximum
