# Supplementary material for: Covalently Doped and Highly Oriented Covalent Organic Framework Thin Films
Source: ACS Nano. 2025 Jul 10;19(28):25720–31. doi: 10.1021/acsnano.5c03065 (PMC12981009; doi:10.1021/acsnano.5c03065)
Supplement: Supplementary file 1 [file nn5c03065_si_001.pdf]

Covalently Doped and Highly Oriented Covalent Organic Framework Thin Films

*Dayanni D. Bhagwandin[a,b], Brian M. Everhart[a,c], Kirt A. Page[a,b,d], Michael A. Altvater[a,b], Yao Yao[e], Rahul Rao[a], Kara Martin[f], Krishnamurthy Mahalingam[a,b], Cheri M. Hampton[a,b], Jonathan Ludwick[a,b], Griffin Roberts[f,g], Drake Austin[a,b], Md Sherajul Islam[a], Ly D. Tran[a], Arthur R. Woll[d], Lawrence F. Drummy[a], Ajit K. Roy[a], Tobin J. Marks[e], Antonio Facchetti[i], Tyson Back[a], Hilmar Koerner[a], Luke A. Baldwin[a], Nicholas R. Glavin[a]**

[a]Air Force Research Laboratory, Materials and Manufacturing Directorate, WPAFB, OH 45433 USA

[b]BlueHalo, Dayton, OH 45432 USA

[c]National Research Council Research Associate, Air Force Research Laboratory, Wright Patterson AFB, OH, 45433, USA

[d]Cornell High Energy Synchrotron Source, Cornell University, Ithaca, New York 14853, USA

[e]Department of Chemistry and the Materials Research Center, Northwestern University, Sheridan Road, Evanston, IL 60208, USA

[f]Air Force Research Laboratory, Aerospace Systems Directorate, WPAFB, OH 45433 USA

[g]University of Dayton Research Institute, 300 College Park, Dayton, OH 45469 USA

[h]School of Materials Science and Engineering, Georgia Institute of Technology Atlanta, GA 30332, USA

List of Figures

Figure S1. Photo of undoped and doped COF thin film on Si/SiO_2. 4
Figure S2. Atomic force microscopy (AFM) images showing edges of undoped and doped COF thin film and underlying substrate. 5
Figure S3. Survey XPS scan of TDA doped COFs (left). Hi-res XPS scan of sulfur region from corresponding films (middle) and hi-res XPS scan of nitrogen region (right). 6
Figure S4. TEM image of and corresponding energy-dispersive X-ray spectroscopy (XEDS) map. 8
Figure S5. Corresponding XEDS Spectrum with sulfur peak. 9
Figure S6. Undoped and doped COF powder. 9
Figure S7. Powder X-Ray diffraction of undoped and doped COF powder. 10
Figure S8. Fourier-transform infrared (FT-IR) spectra of TDA, PDA, TAPB starting monomers, and undoped and doped COF powders. 10
Figure S9. Fourier-transform infrared (FT-IR) spectra of undoped and doped COF powder with highlighted differences. 11
Figure S10. Raman spectra of TDA, PDA, TAPB starting monomers, and undoped and doped COF powders. 12
Figure S11. ^{1}H-NMR of acid digested doped COF powder. 13
Figure S12. GIWAXS of doped COF film series with percent of aldehyde TDA and PDA initially added to the reaction. 14
Figure S13. Typical 2D scattering pattern from a doped COF film demonstrating the vertical and azimuthal scattering. 14
Figure S14. TEM of undoped TAPB-PDA COF 16
Figure S15. TEM of undoped TAPB-PDA COF 17
Figure S16. TEM of undoped TAPB-PDA COF 18
Figure S17.TEM of doped COF synthesized with 50% TDA and 50% PDA 19
Figure S18. TEM of doped COF synthesized with 50% TDA and 50% PDA 20
Figure S19. TEM of doped COF synthesized with 50% TDA and 50% PDA 21
Figure S20. TEM of doped COF synthesized with 50% TDA and 50% PDA 22
Figure S21. GIWAXS of 24-hour growth TAPB-PDA COF film (left) after annealing, (middle) after one year, and (right) after annealing a second time after one year 23
Figure S22. GIWAXS of 24-hour growth doped COF film (left) after annealing, (middle) after one year, and (right) after annealing a second time after one year. 23
Figure S23. GIWAXS of 8-month old, 3-week growth doped COF sample (left) before sonication and (right) after sonication. 24
Figure S24. Vertex and linker molecule HOMO and LUMO states. 25
Figure S25. Tight-binding model. a) DFT-calculated conduction bands of the PDA COF (red crosses) and Kagome tight binding model fit (dashed lines). b) DFT-calculated conduction bands of the TDA-doped COF (red crosses) and symmetry broken-Kagome tight binding model fit (dashed lines). c) DFT-calculated conduction bands of the PDA COF (red crosses) and honeycomb-trimer lattice tight binding model fit (dashed lines) d) DFT-calculated conduction bands of the TDA-doped COF (red crosses) and symmetry broken honeycomb-trimer lattice tight binding model fit (dashed lines). 26

Figure S26. Path through the Brillouin zone (BZ) 27
Figure S27. XPS scans of doped COF films used for construction of the band diagram. ... 27
Figure S28. UV-Vis (a) and XPS (b) measurements of band gap and work function, respectively. Figures (c) and (d) present the average band gap and work function values calculated from the data in (a) and (b). 29

List of Tables

Table S1. Equivalents of TDA and PDA used to synthesize series of films. 4
Table S2. XPS Peak fitting raw peak areas for spectra in Figure S3. 7
Table S3. Raman Peaks and Intensities for Doped COF Film Series 24
Table S4. Raw deconvoluted XPS peak areas corresponding to the N1s and S2p spectra in **Figure S27**........ 28
Table S5. Raw deconvoluted XPS peak positions corresponding to the N1s spectra in **Figure S27** and peak areas in **Table S4**. 28
Table S6. Raw deconvoluted XPS peak areas corresponding to the C1s spectra in **Figure S27**. 28
Table S7. Raw deconvoluted XPS peak positions corresponding to the C1s spectra in **Figure S27** and peak areas in **Table S6**. 29
Table S8. Experimentally Determined Band Diagram Parameters 29

Experiment	TDA [moles]	PDA [moles]	TDA Solution [mL][a)]	PDA Solution [mL][b)]
A	0.0056	0.0504	0.8	7.2
B	0.0112	0.0448	1.6	6.4
C	0.0224	0.0336	3.2	4.8
D	0.0336	0.0224	4.8	3.2
E	0.0448	0.0112	6.4	1.6
F	0.056	0	8	0
[a)]TDA Solution: 0.98 mg/mL; [b)]PDA Solution: 0.94 mg/mL				

Table S1. Equivalents of TDA and PDA used to synthesize series of films.

Solutions were prepared by adding the aldehyde to methylene chloride and sonicating.

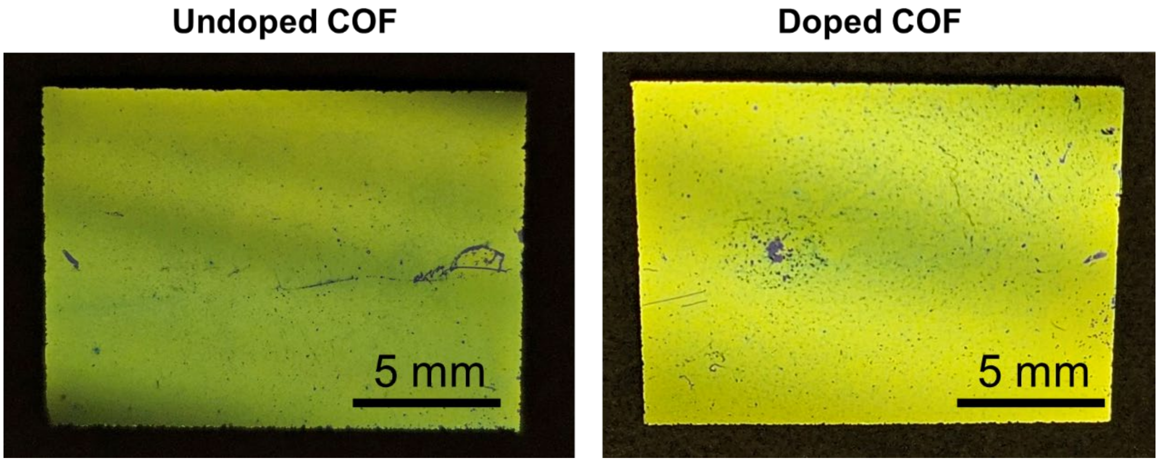

Figure S1. Photo of undoped and doped COF thin film on Si/SiO_2.

The doped COF film shown in **Figure S1** and was synthesized using a 1:9 TDA to PDA ratio.

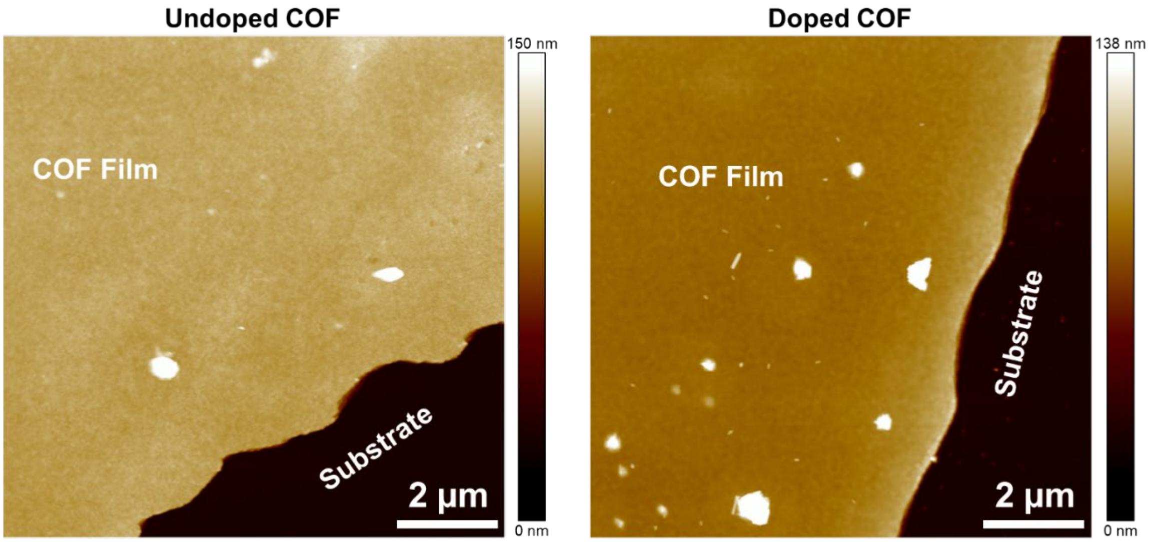

Figure S2. Atomic force microscopy (AFM) images showing edges of undoped and doped COF thin film and underlying substrate.

Film thickness measurements based of AFM images reveal that the undoped COF film has a thickness around 84 nm while the doped COF film has a thickness around 60 nm. The undoped COF film had a roughness (Rq) of about 2 nm with clusters of COF particles (white spots) about 100 nm in height. The doped COF film also had a roughness of 2 nm with more COF particles throughout ranging between 100 and 200 nm in height. The undoped COF film was synthesized using a 1:9 ratio of TDA to PDA and TDA presence was confirmed using Raman spectroscopy. For the undoped film, the scan rate was 0.957 Hz and the drive amplitude was 1079.4 mV. For the doped film, the scan rate was 0.957 Hz and the drive amplitude was 751.2 mV.

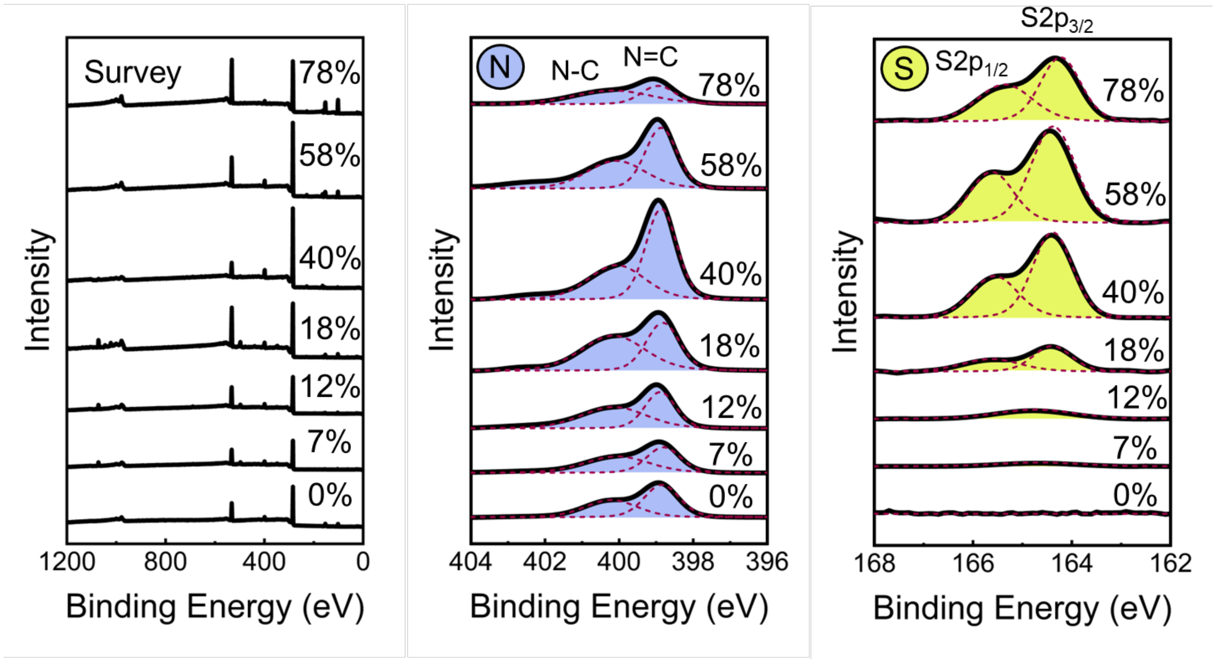

Figure S3. Survey XPS scan of TDA doped COFs (left). Hi-res XPS scan of sulfur region from corresponding films (middle) and hi-res XPS scan of nitrogen region (right).

Deconvoluted peak fits are shown as dashed red lines in the XPS.

As mentioned in the main body of the work, the extent of TDA linkers in the film can be estimated by comparing the sulfur and nitrogen ratios as in the idealized COF structure. When determining the percentage of TDA incorporation into the TAPB-PDA COF, the following equation was used:

$$\frac{2 * Adjusted\ S\ 2p\ Peak\ Area}{Adjusted\ N\ 1s\ Peak\ Area + Adjusted\ S\ 2p\ Peak\ Area} * 100\% \qquad \textbf{Equation 1}$$

Where

$$Adjusted\ S\ 2p\ Peak\ Area = \frac{\left(S\ 2p_{\frac{1}{2}} + S\ 2p_{\frac{3}{2}}\right)}{RSF_{S2p}} \qquad \textbf{Equation 2}$$

And

$$Adjusted\ N\ 1s\ Peak\ Area = \frac{(N\ 1s_{N=C} + N\ 1s_{N-C})}{RSF_{N1s}} \qquad \textbf{Equation 3}$$

The relative sensitivity factors (RSF) for S 2p and N 1s are 1.677 and 1.80, respectively. Because every linker is connected to two imine nitrogens, the equation multiplies the adjusted S 2p peak area by 2 to determine the mole percent of TDA incorporation.

Survey scan measurements were performed using a pass energy of 89.05 eV, a step size of 1.0eV, and a 100ms dwell time, while VBM measurements, high-resolution elemental

scans (S2p, N1s, C1s), and work function measurements were all performed using a pass energy of 20 eV, a step size of 0.1eV, and 100ms dwell time.

	Raw Peak Area				
COF Film	N=C	N-C	S2p3/2	S2p1/2	TDA %
1a	449	351	0	0	0
2a	342	414	26.6	0	7.3
3a	440	551	60.9	0	12.4
4a	634	880	81.2	59.8	18.2
5a	1124	771	282.7	162.3	40.3
6a	764	643	353.3	185.6	58.3
7a	246	420	220.8	185.6	77.6

Table S2. XPS Peak fitting raw peak areas for spectra in Figure S3.

The TEM sample used for the analysis in **Figure S4** and **Figure S5** was prepared using the same method used for thin film growth, but instead the Si substrate was replaced with a TEM grid. This sample was prepared using a 1:1 ratio of PDA to TDA.

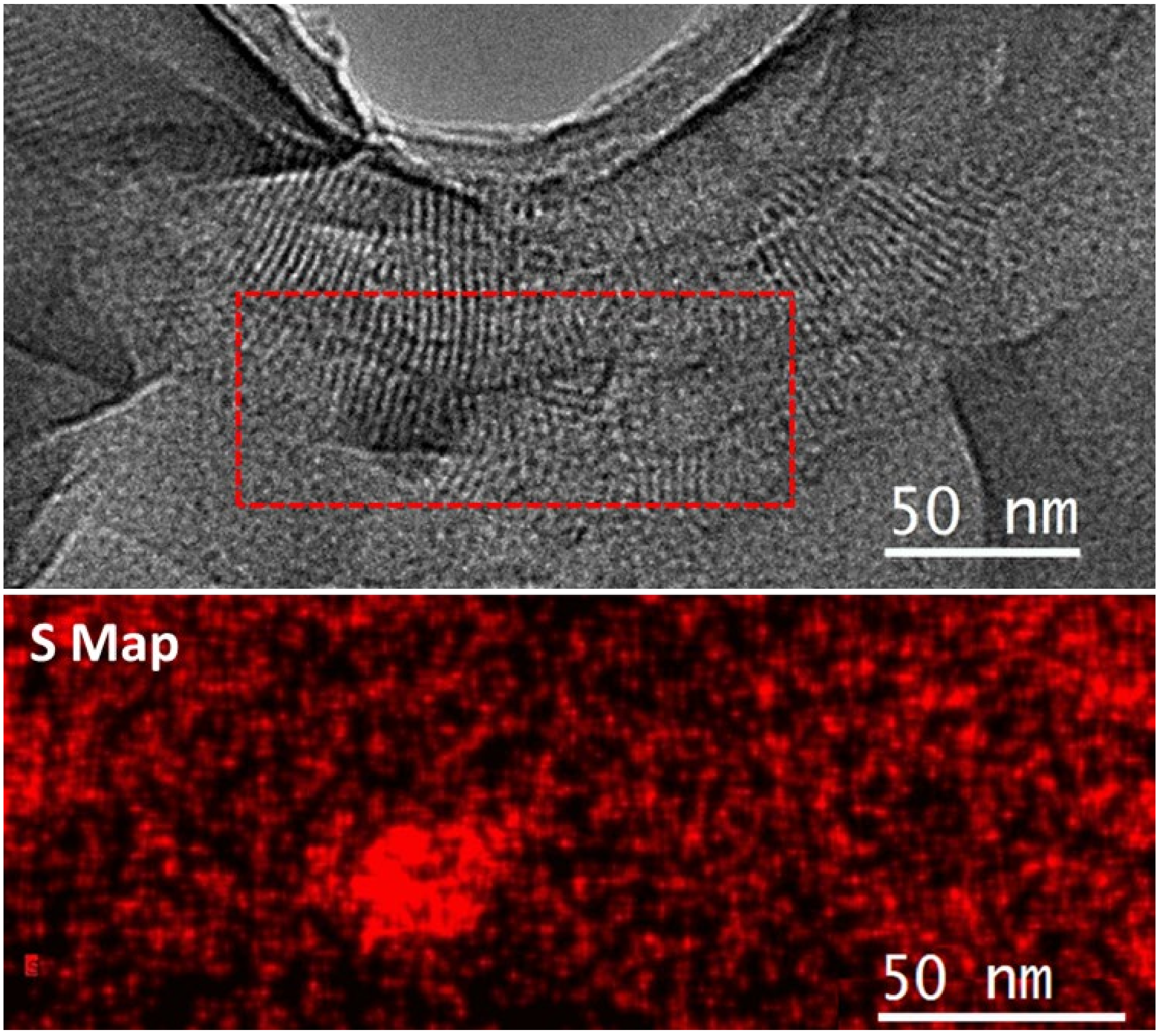

Figure S4. TEM image of and corresponding energy-dispersive X-ray spectroscopy (XEDS) map.

The crystalline region in the TEM image is outlined in the red box and the sulfur map below shows the area contained in the red box. The XEDS spectrum below represents the entire sulfur map.

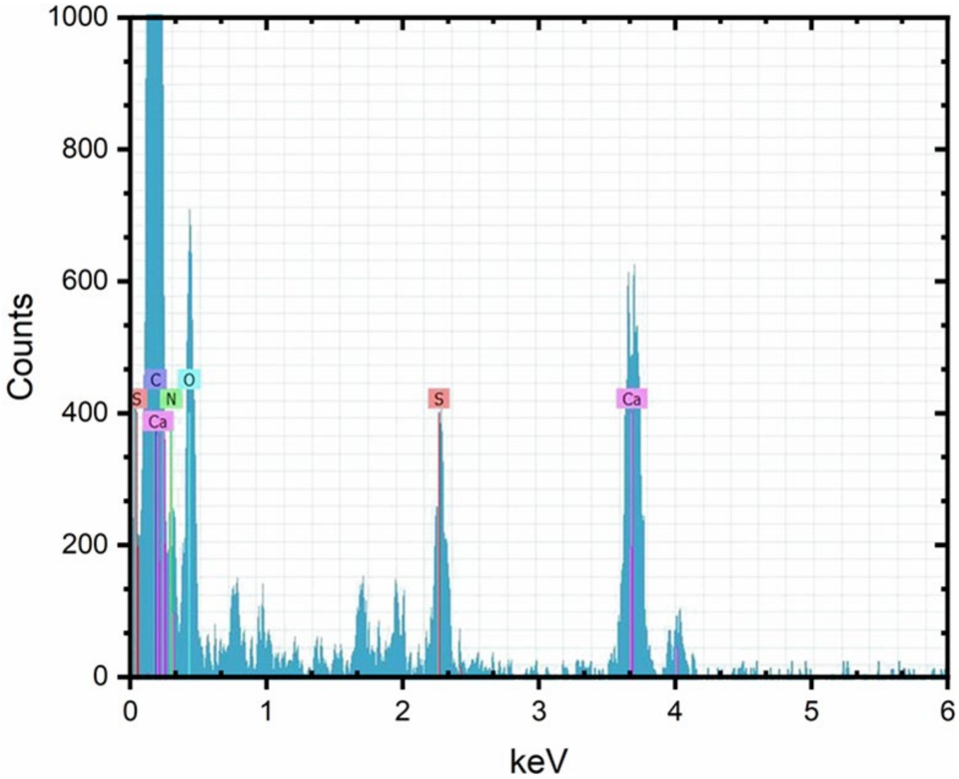

Figure S5. Corresponding XEDS Spectrum with sulfur peak.

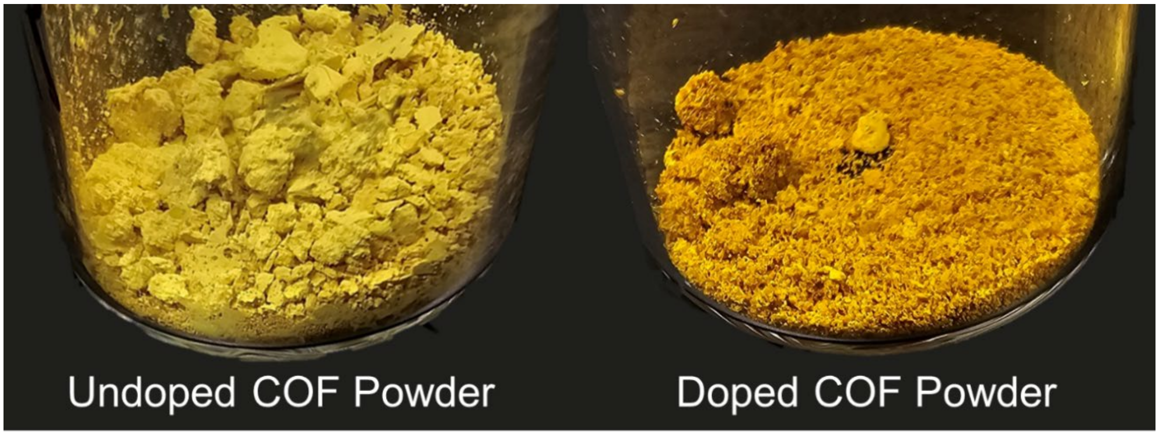

Figure S6. Undoped and doped COF powder.

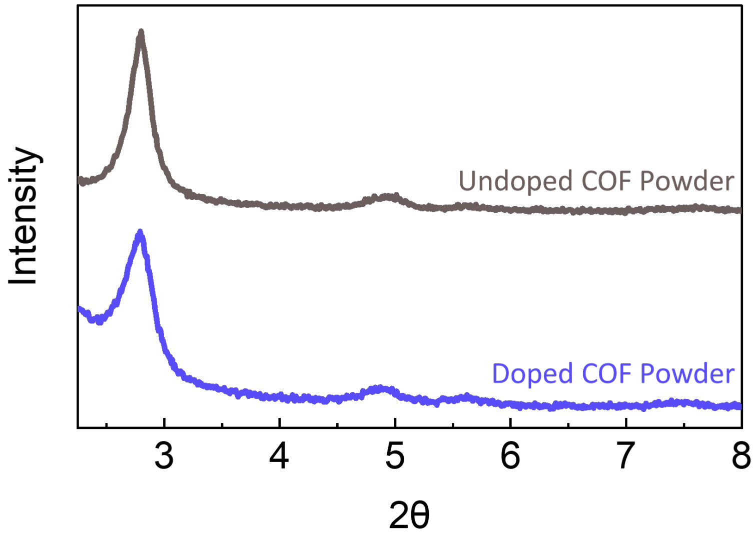

Figure S7. Powder X-Ray diffraction of undoped and doped COF powder.

Powder X-Ray diffraction of both samples show the peak associated with the repeat distance of the pore in the (100) plane at 2.8 2θ which corresponds to a d-spacing of 3.2 nm. Additionally peaks associated with repeat distances in the [200] plane at 4.9 2θ and the [210] plane at 5.6 2θ with d-spacings of 1.8 and 1.6 nm, respectively.

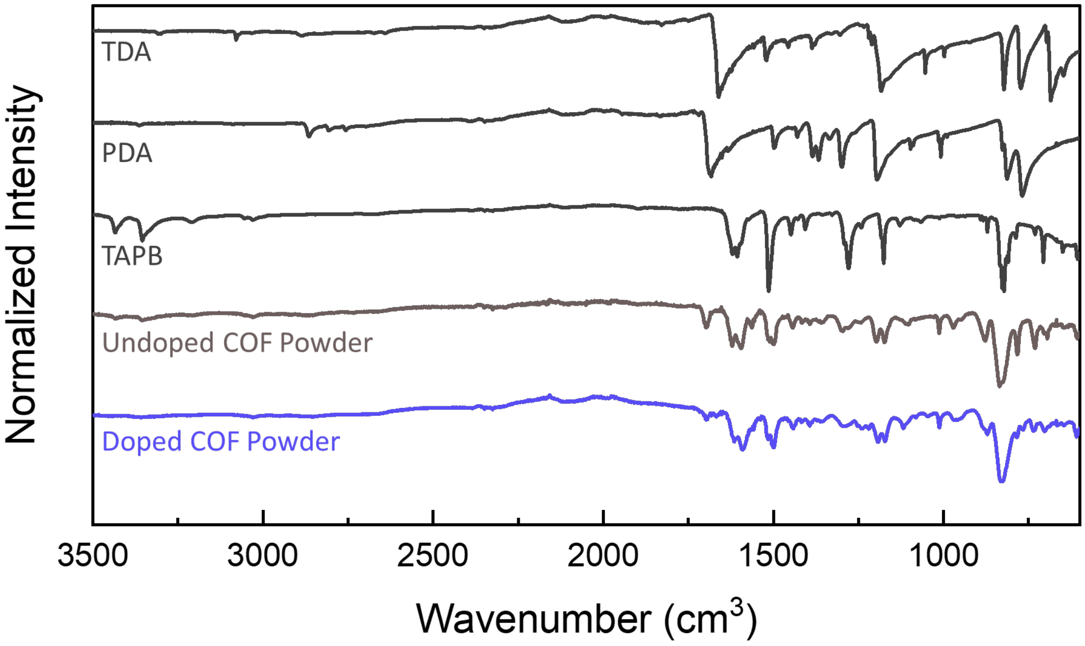

Figure S8. Fourier-transform infrared (FT-IR) spectra of TDA, PDA, TAPB starting monomers, and undoped and doped COF powders.

The FT-IR spectra clearly illustrate the chemical transformations occurring during COF formation from the starting materials: TAPB, TDA, and PDA. TAPB, possessing amine functional groups, exhibits characteristic N-H stretching vibrations in the 3300-3500 cm^{-1} region. Conversely, TDA and PDA, both containing aldehyde groups, show characteristic C=O stretching vibrations typically around 1700 cm^{-1}. Upon COF formation in the undoped sample (TAPB-PDA), the near disappearance of the N-H stretching from TAPB and the C=O stretching from PDA, coupled with the emergence of a strong C=N stretching band around 1620-1680 cm^{-1},[1] confirms the successful formation of imine linkages, which constitute the backbone of the COF framework. In the doped COF, where a thiophene-containing linker is incorporated alongside TAPB and PDA, the spectra exhibit similar imine formation.

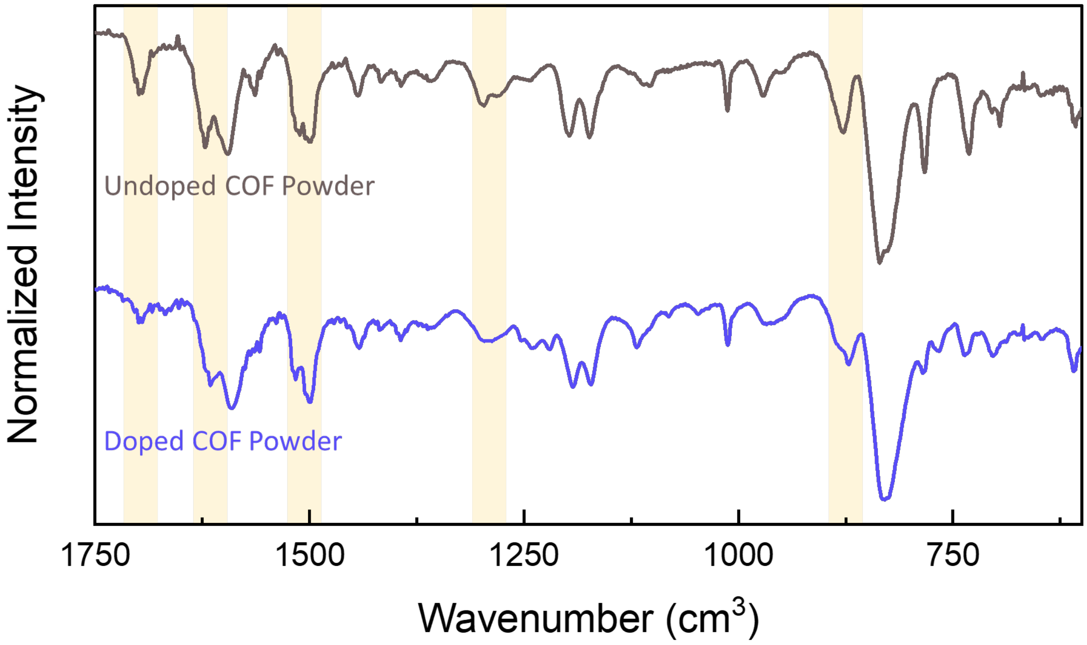

Figure S9. Fourier-transform infrared (FT-IR) spectra of undoped and doped COF powder with highlighted differences.

The doped COF also presents additional or shifted peaks, particularly below the 1500 cm^{-1} region, attributable to the vibrational modes of the thiophene unit (e.g., C-S-C stretching, C-H ring stretching of aromatic protons).[2] These distinct spectral features confirm the successful integration of the thiophene linker into the COF structure, resulting in a chemically distinct material compared to the undoped TAPB-PDA COF.

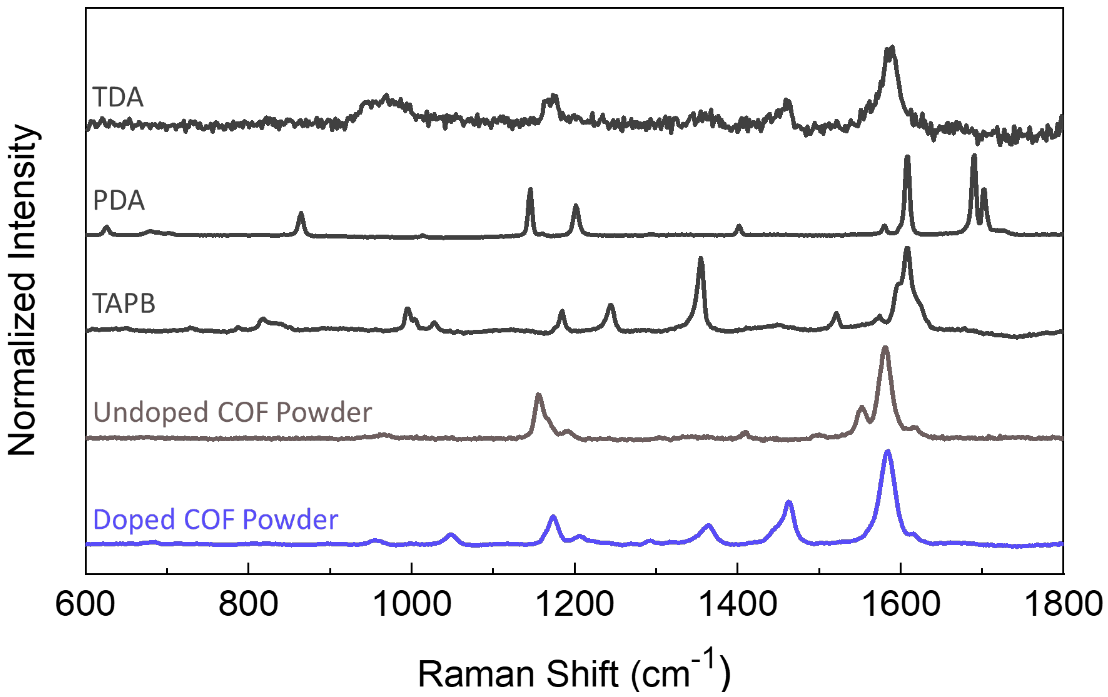

Figure S10. Raman spectra of TDA, PDA, TAPB starting monomers, and undoped and doped COF powders.

Raman spectra associated with both the samples show clear differences in peak patterns. It also shows that no starting material remains in the films. In the doped sample, the peak at 1154 cm^{-1} disappears, while the intensity ratio of the 1170/1165 cm^{-1} peaks increases compared to the undoped sample. Additionally, peaks at 1360 cm^{-1}, 1450 cm^{-1}, and 1464 cm^{-1} can be seen in the doped sample. The peak at 1590 cm^{-1} also shifts to lower wavenumbers and the intensity ratios of the 1590 cm^{-1} and 1565 cm^{-1} peaks also increases in the doped sample. From overall analysis of the Raman spectra and comparison to the doped thin film series, it is estimated that the doped COF powder sample contains approximately 50% of linkers with TDA.

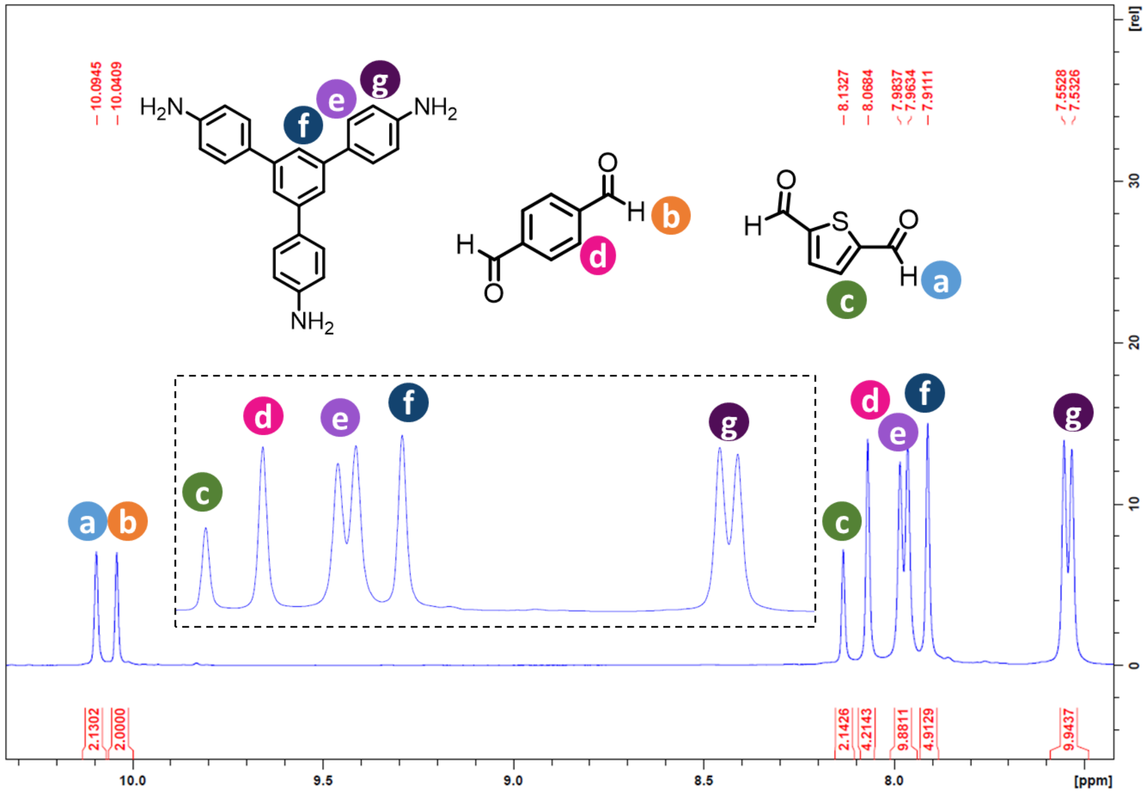

Figure S11. ^{1}H-NMR of acid digested doped COF powder.

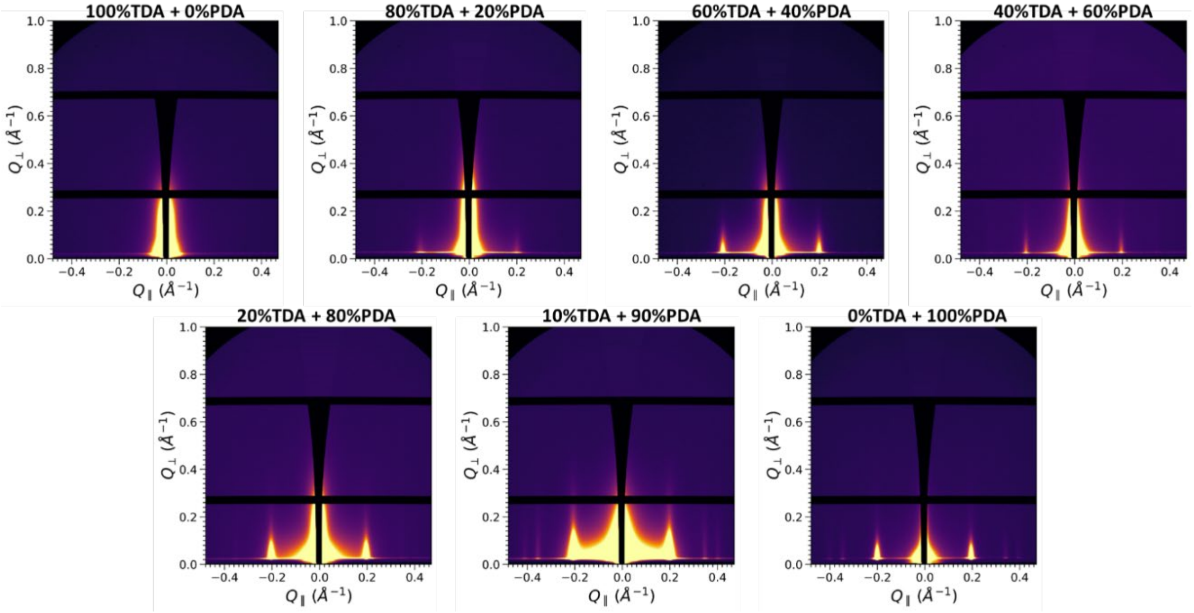

Figure S12. GIWAXS of doped COF film series with percent of aldehyde TDA and PDA initially added to the reaction.

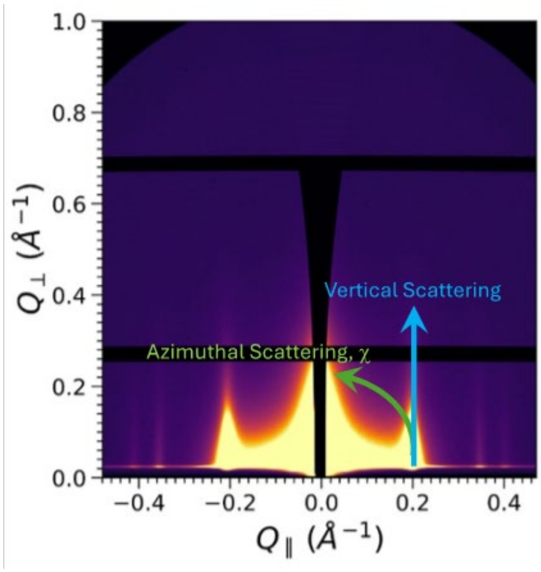

Figure S13. Typical 2D scattering pattern from a doped COF film demonstrating the vertical and azimuthal scattering.

The orientation of the crystalline planes, and therefore the COF layers, can be determined by the nature of the scattering in the 2D scattering images. The distribution of the scattering intensity around the azimuthal angle, c, is an indication of both the direction of orientation

relative to the substrate, but also the degree to which those domains are oriented (**Figure S13**).

The Herman's orientation parameter (HOP) is typically determined by analyzing the scattering intensity as a function of the azimuthal angle, c, and is given by the following equation[3]

$$HOP = f = \frac{3\langle\cos^2\chi\rangle - 1}{2}$$ **Equation 4**

Where:

$$\langle\cos^2\chi\rangle = \frac{\int_0^{\frac{\pi}{2}} \mathrm{I}(\chi)\cos^2\chi\sin\chi\, d\chi}{\int_0^{\frac{\pi}{2}} \mathrm{I}(\chi)\sin\chi\, d\chi}$$ **Equation 5**

The HOP can assume a value from -0.5 to 1. In this study, a value of -0.5 would indicate that the COF layers are perfectly oriented perpendicular to the substrate and a value of 1 would indicate that the COF layers are oriented perfectly parallel to the substrate. While the HOP is a useful parameter in characterizing the orientation in these systems, the lack of scattering in the azimuthal direction beyond the width of the vertical peaks does not allow one to use the HOP to quantify the orientation in these samples.

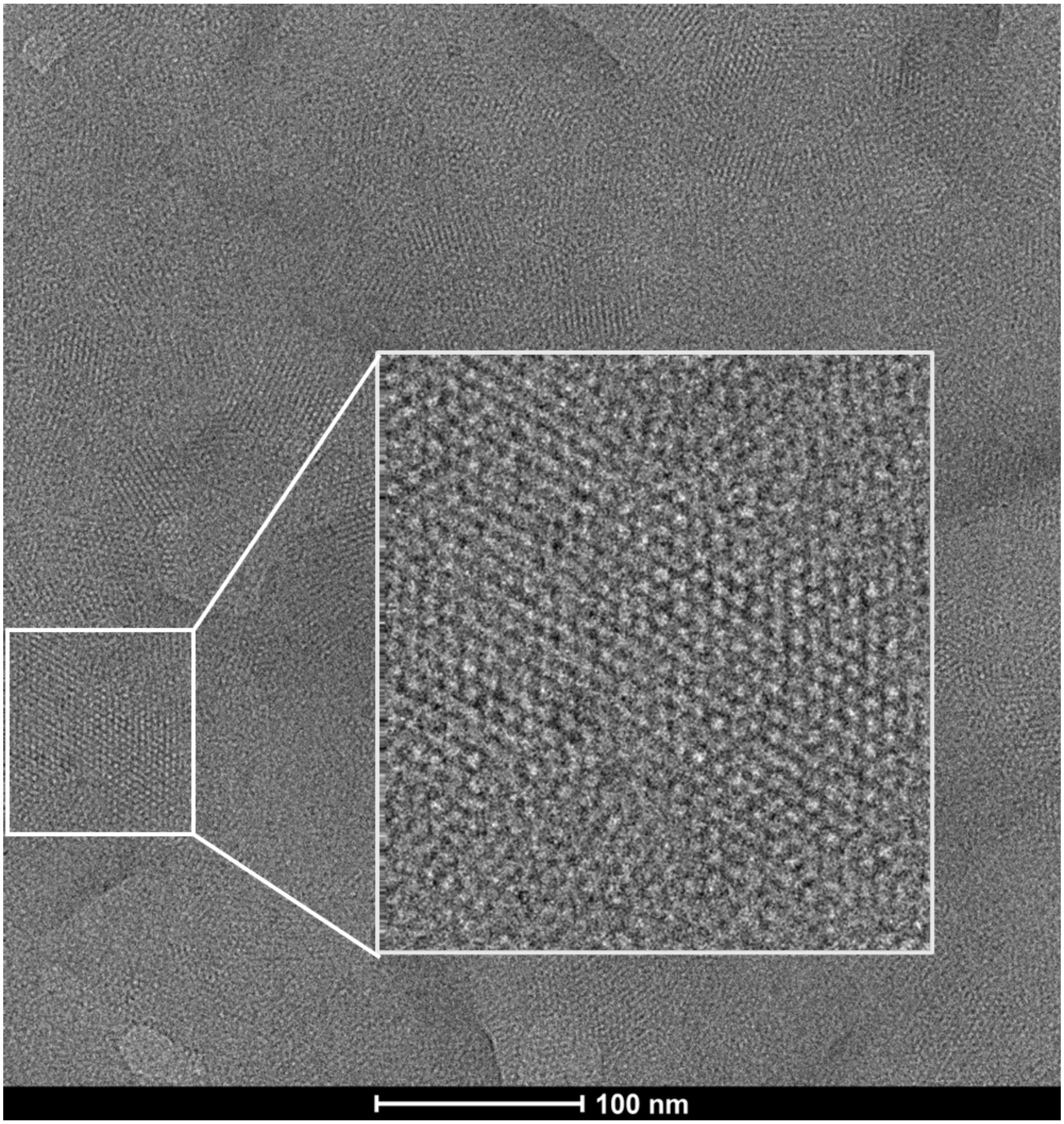

Figure S14. TEM of undoped TAPB-PDA COF

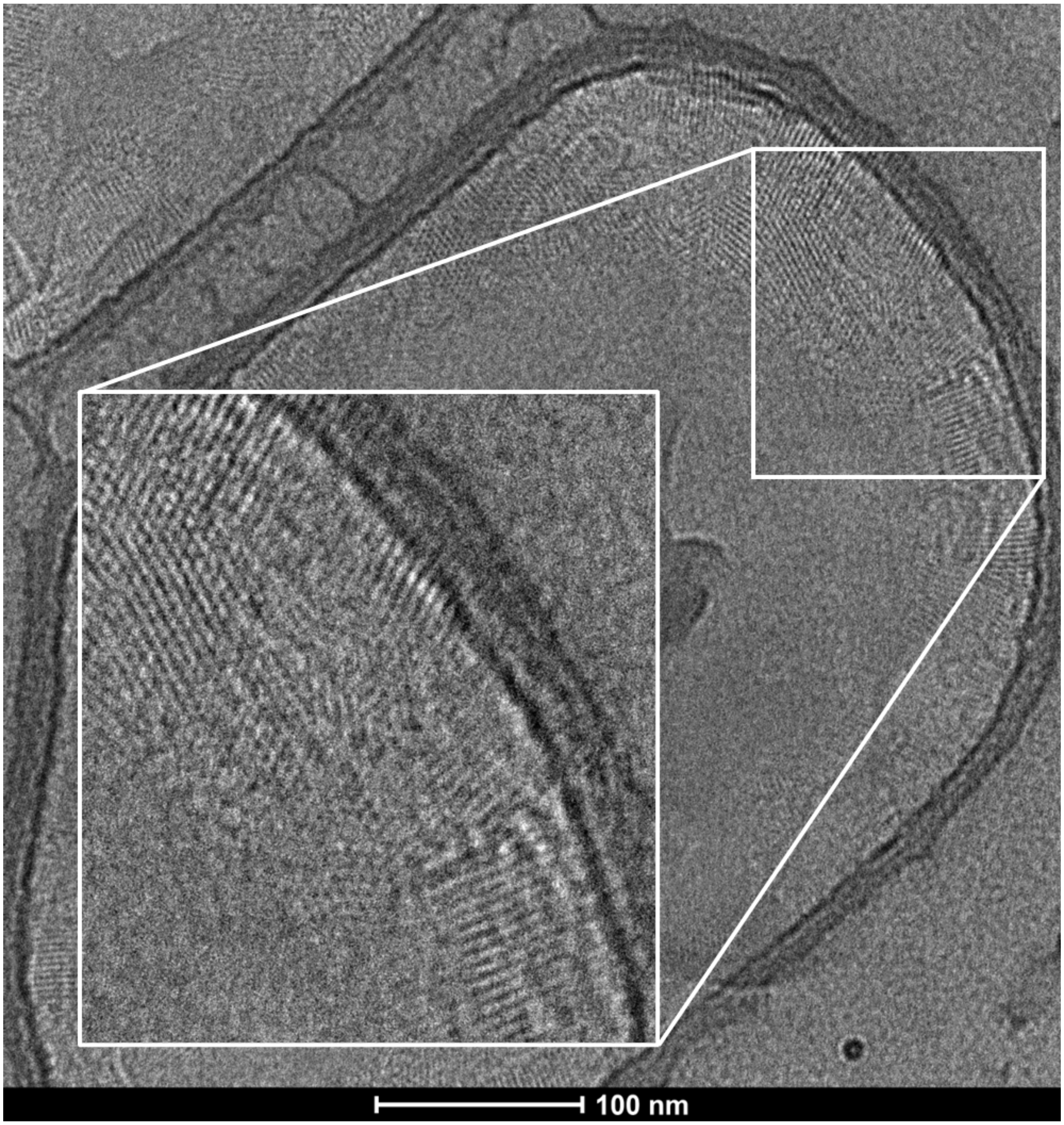

Figure S15. TEM of undoped TAPB-PDA COF

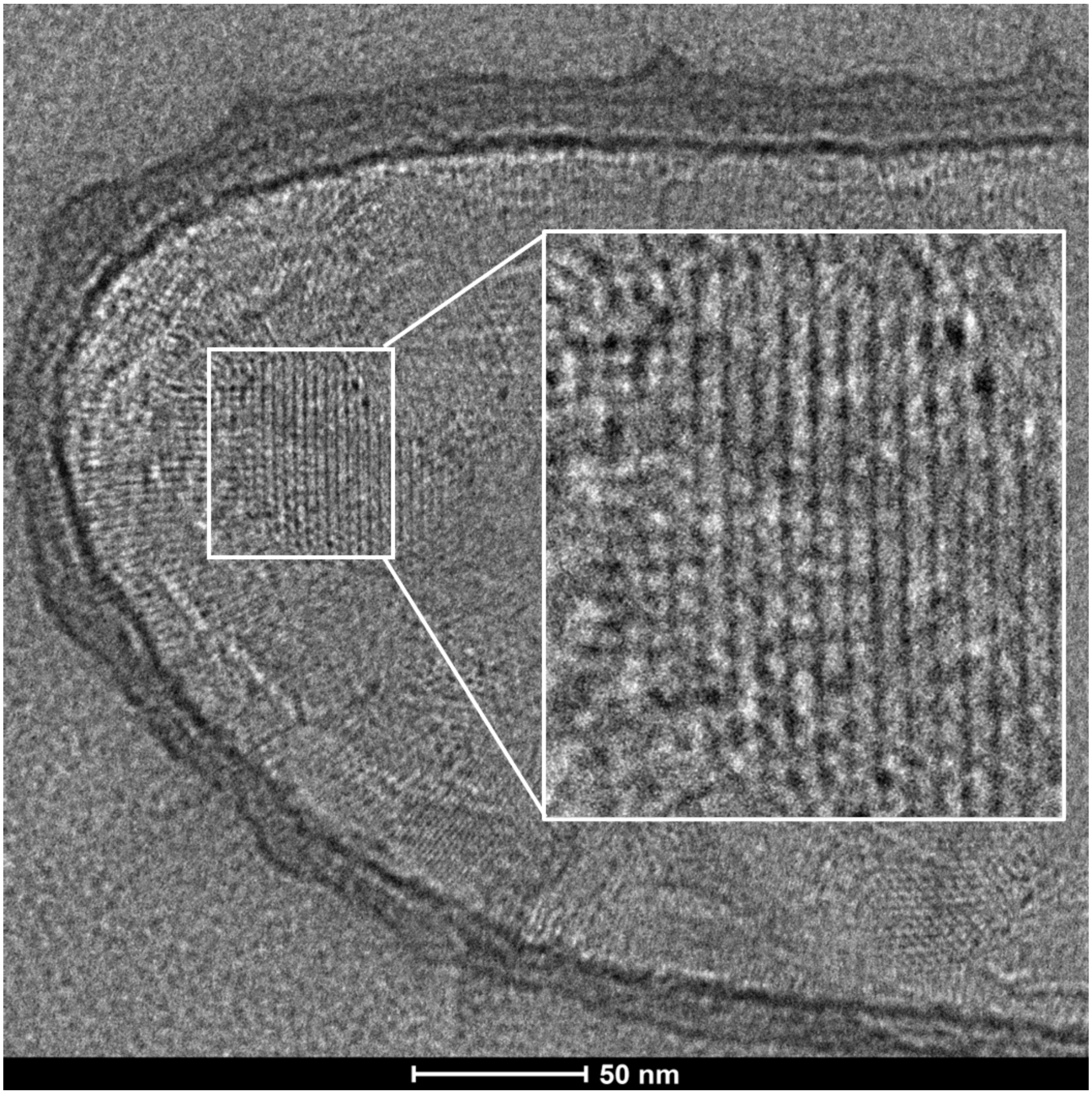

Figure S16. TEM of undoped TAPB-PDA COF

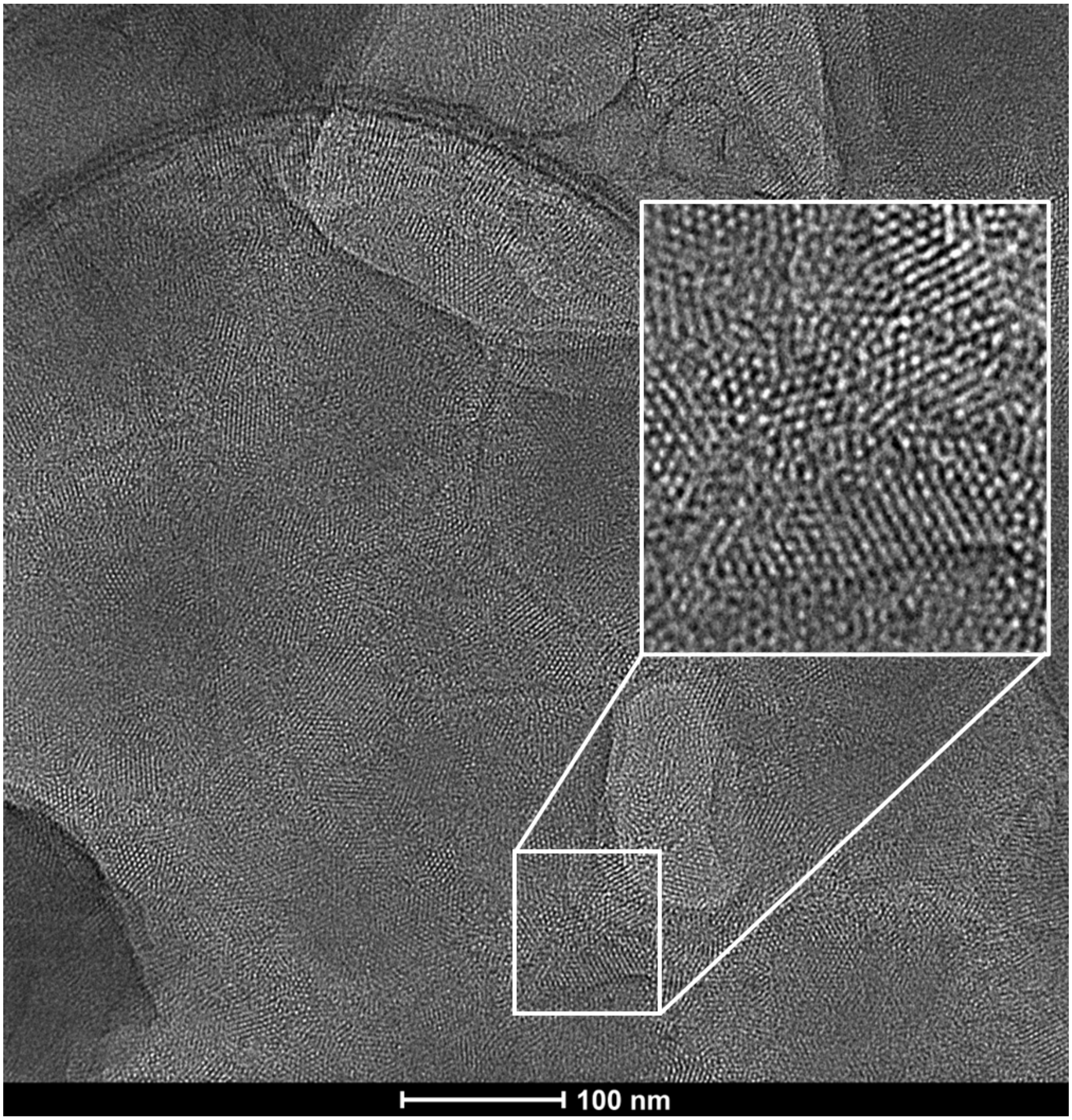

Figure S17.TEM of doped COF synthesized with 50% TDA and 50% PDA

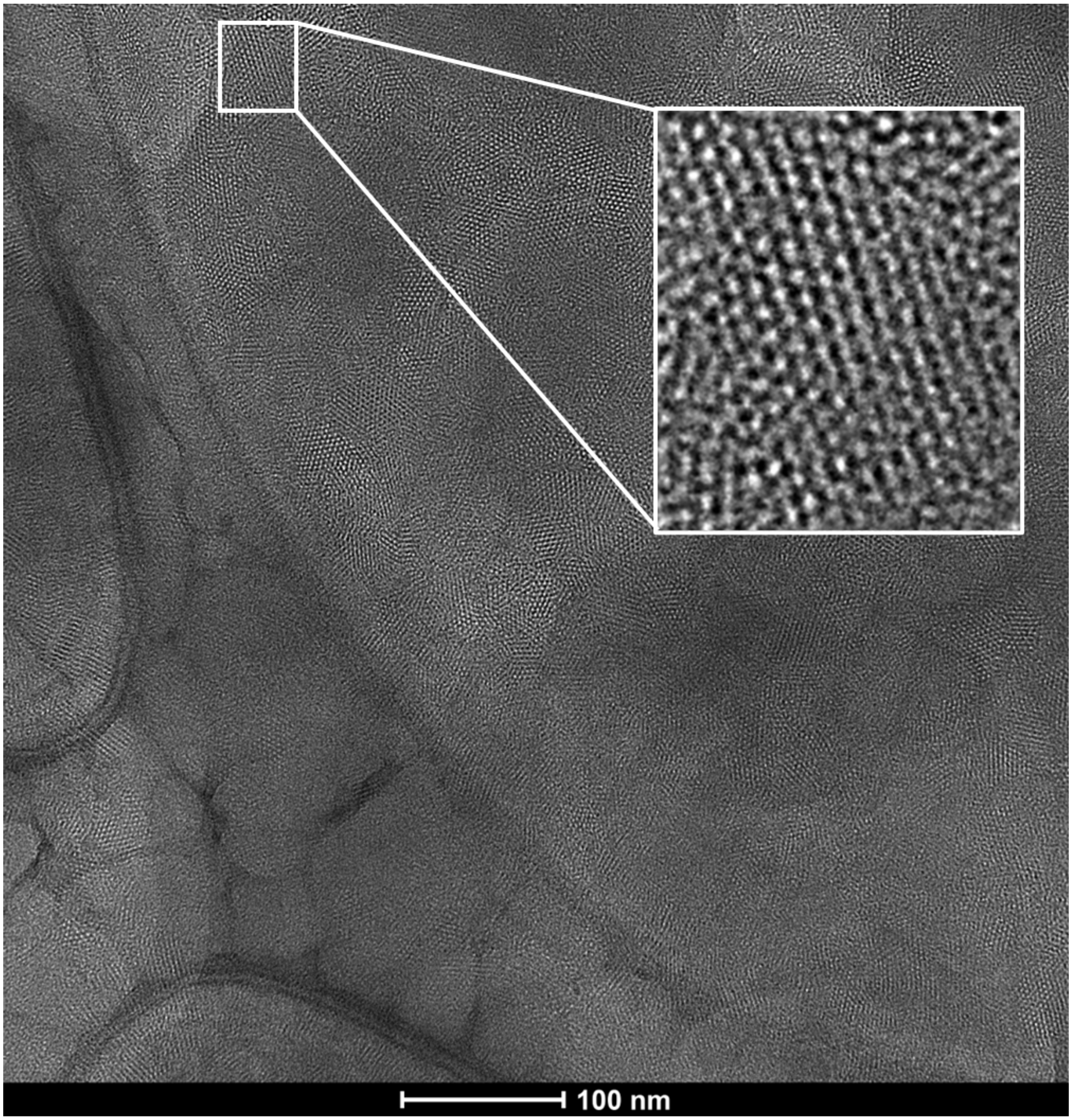

Figure S18. TEM of doped COF synthesized with 50% TDA and 50% PDA

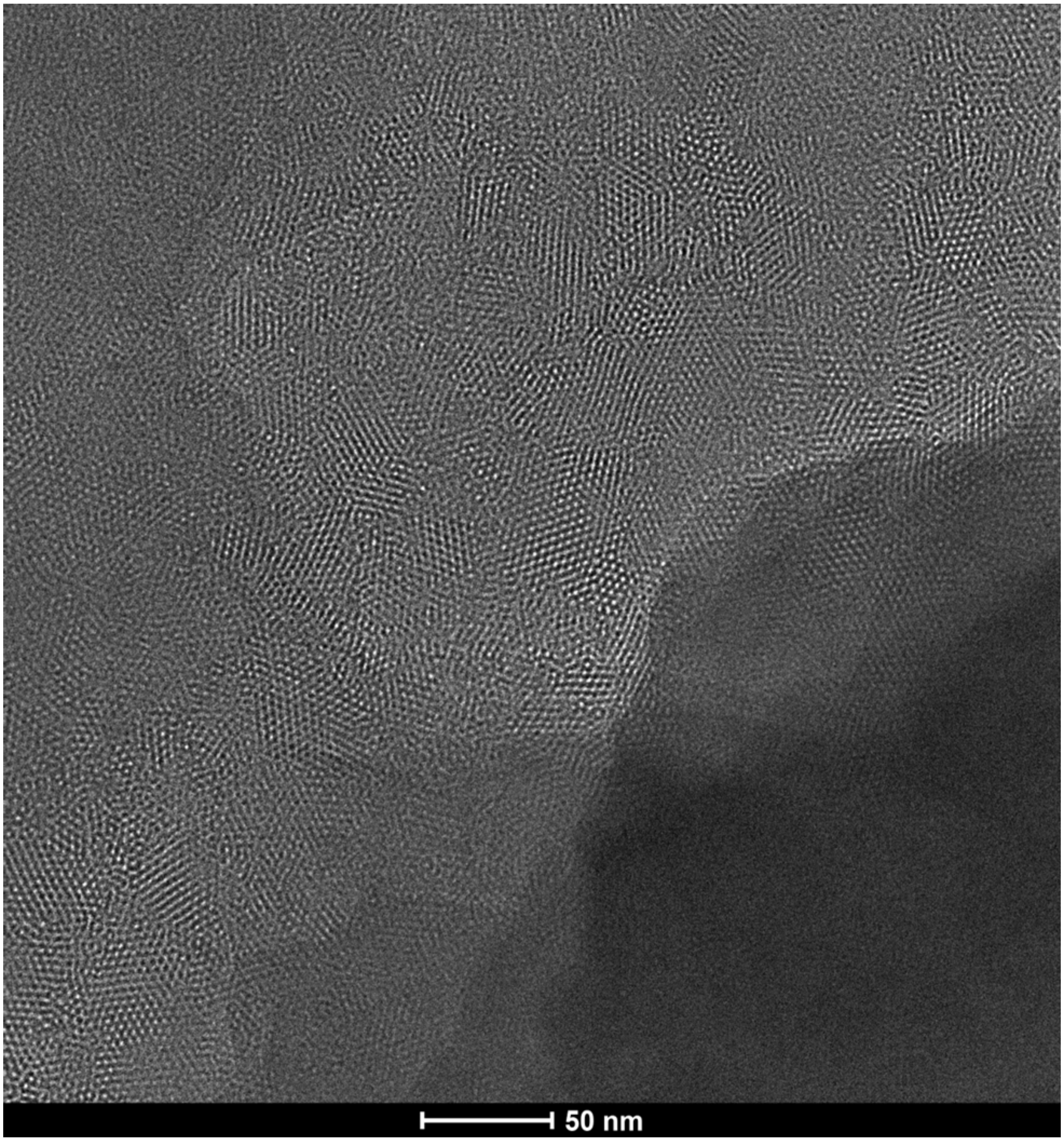

Figure S19. TEM of doped COF synthesized with 50% TDA and 50% PDA

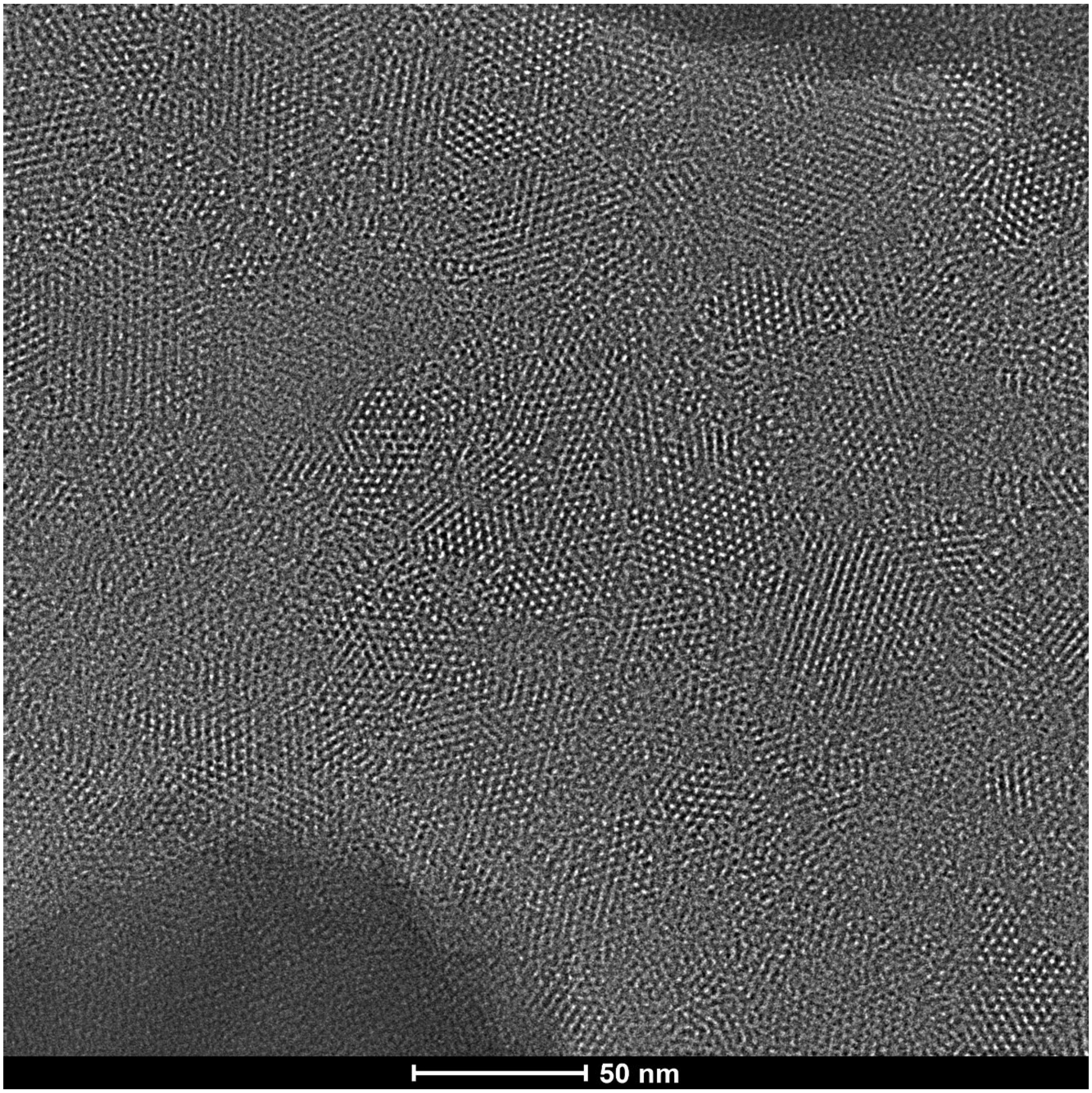

Figure S20. TEM of doped COF synthesized with 50% TDA and 50% PDA

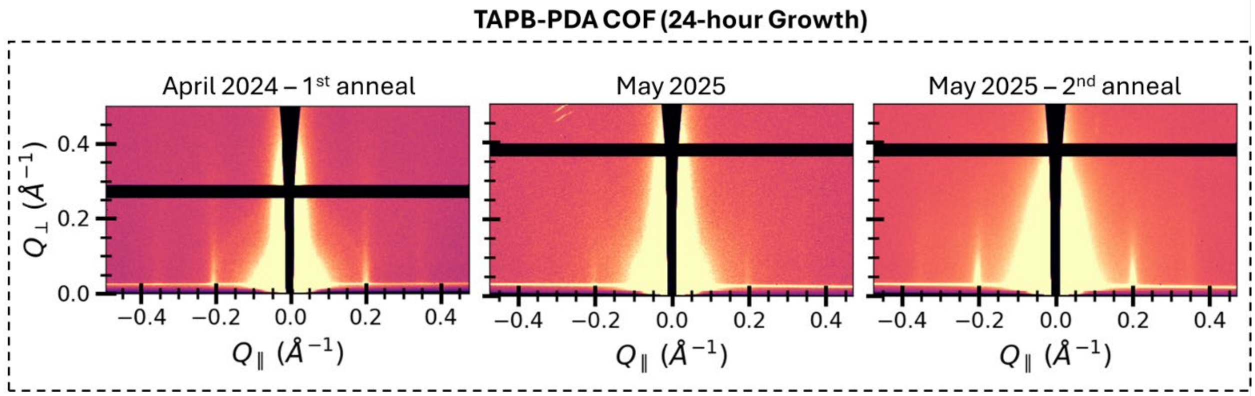

Figure S21. GIWAXS of 24-hour growth TAPB-PDA COF film (left) after annealing, (middle) after one year, and (right) after annealing a second time after one year

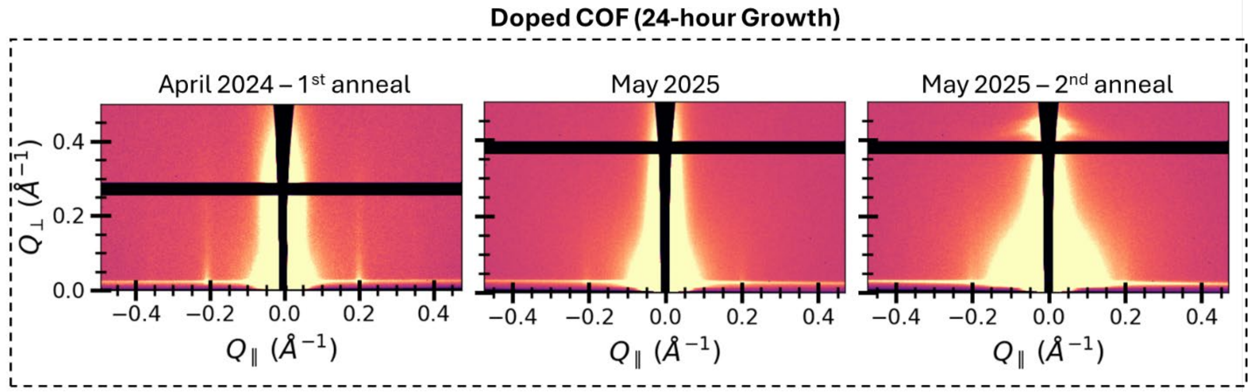

Figure S22. GIWAXS of 24-hour growth doped COF film (left) after annealing, (middle) after one year, and (right) after annealing a second time after one year.

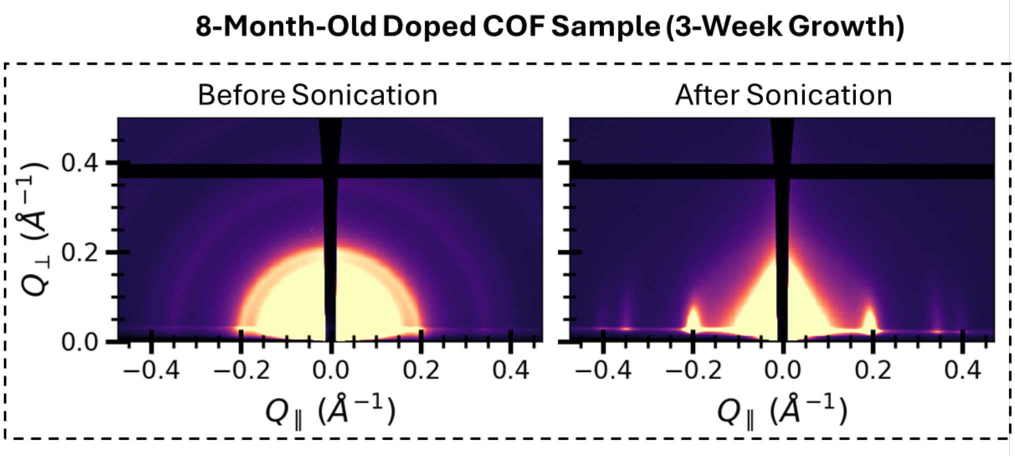

Figure S23. GIWAXS of 8-month old, 3-week growth doped COF sample (left) before sonication and (right) after sonication.

	Peak 1160 cm^{-1}		Peak 1450 cm^{-1}		Peak 1560 cm^{-1}		Peak 1590 cm^{-1}	
	Shift (cm^{-1})	Intensity	Shift (cm^{-1})	Intensity	Shift (cm^{-1})	Intensity	Shift (cm^{-1})	Intensity
0%	1164	0.33486	-	-	1565	0.38052	1591	1
7%	1164	0.51612	-	-	1561	0.44443	1591	1
12%	1164	0.46155	-	-	1559	0.38031	1591	1
18%	1164	0.40231	-	-	1559	0.33416	1589	1
40%	1166	0.34047	1457	0.38192	1566	0.43387	1588	1
58%	1169	0.36618	1462	0.52385	1568	0.39338	1586	1
77%	1169	0.28378	1462	0.55038	-	-	1584	1

Table S3. Raman Peaks and Intensities for Doped COF Film Series

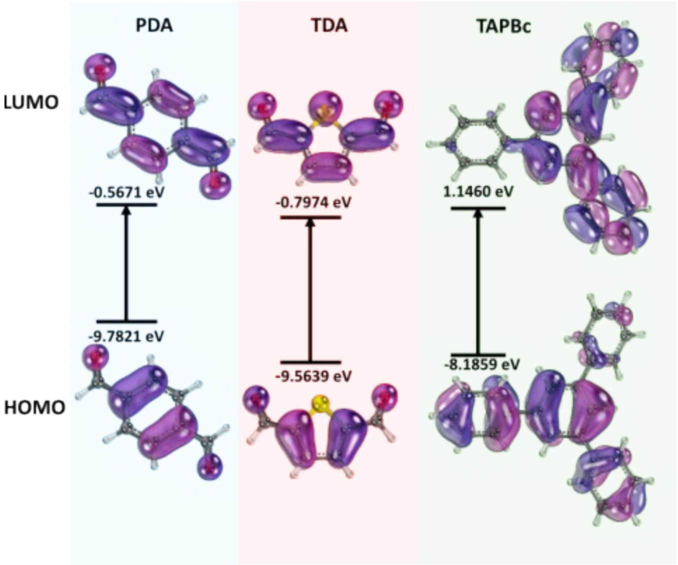

Figure S24. Vertex and linker molecule HOMO and LUMO states.

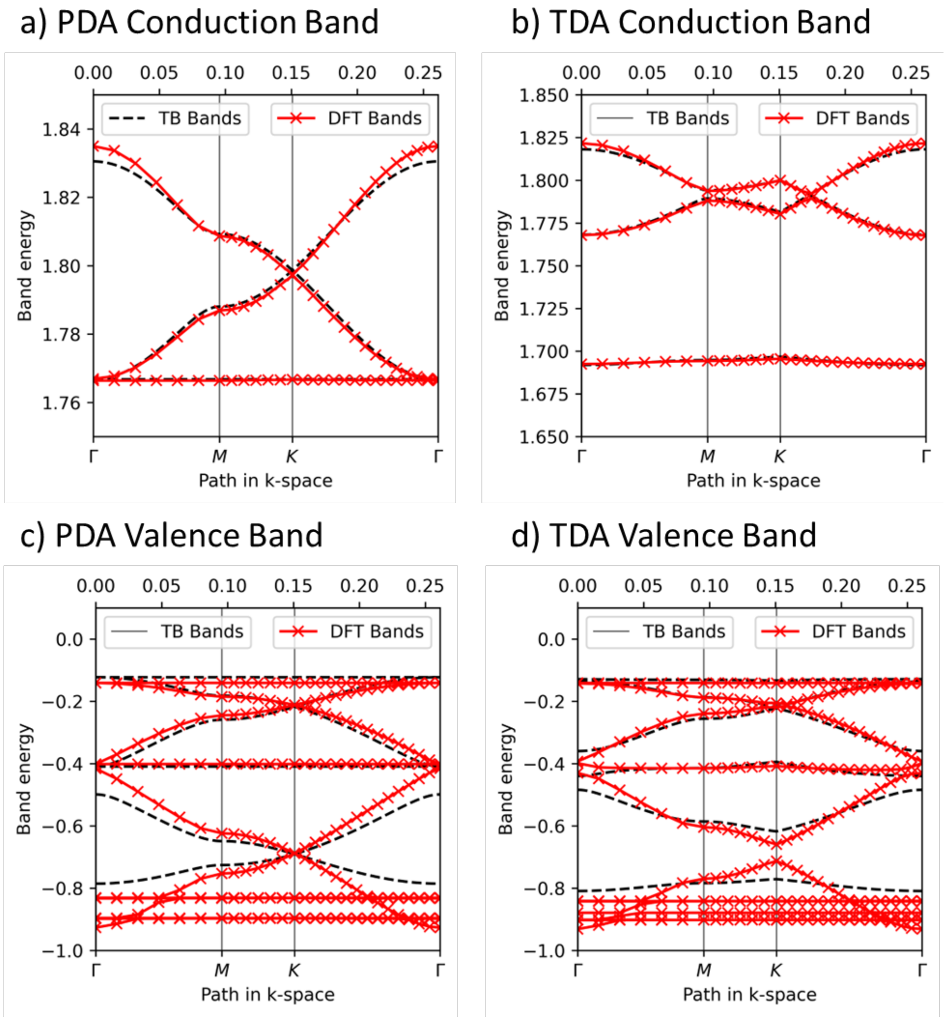

Figure S25. Tight-binding model. a) DFT-calculated conduction bands of the PDA COF (red crosses) and Kagome tight binding model fit (dashed lines). b) DFT-calculated conduction bands of the TDA-doped COF (red crosses) and symmetry broken-Kagome tight binding model fit (dashed lines). c) DFT-calculated conduction bands of the PDA COF (red crosses) and honeycomb-trimer lattice tight binding model fit (dashed lines) d) DFT-calculated conduction bands of the TDA-doped COF (red crosses) and symmetry broken honeycomb-trimer lattice tight binding model fit (dashed lines).

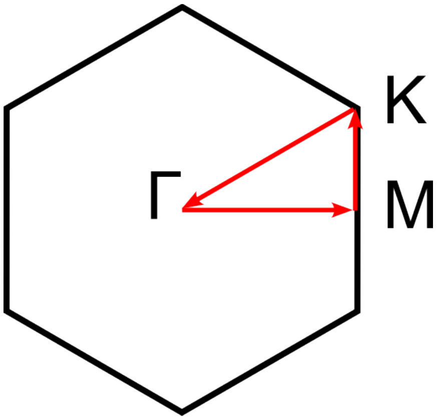

Figure S26. Path through the Brillouin zone (BZ)

This is the path through the Brillouin zone (BZ) along which the band structures are plotted. The hexagon represents the BZ, the arrows show the path, and the letters label the high symmetry points marked in the band structure plots (Gamma is in the middle, M is the middle of the edge, and K is the corner).

The figure below shows the XPS scans used for the construction of the band diagram in **Figure 6**.

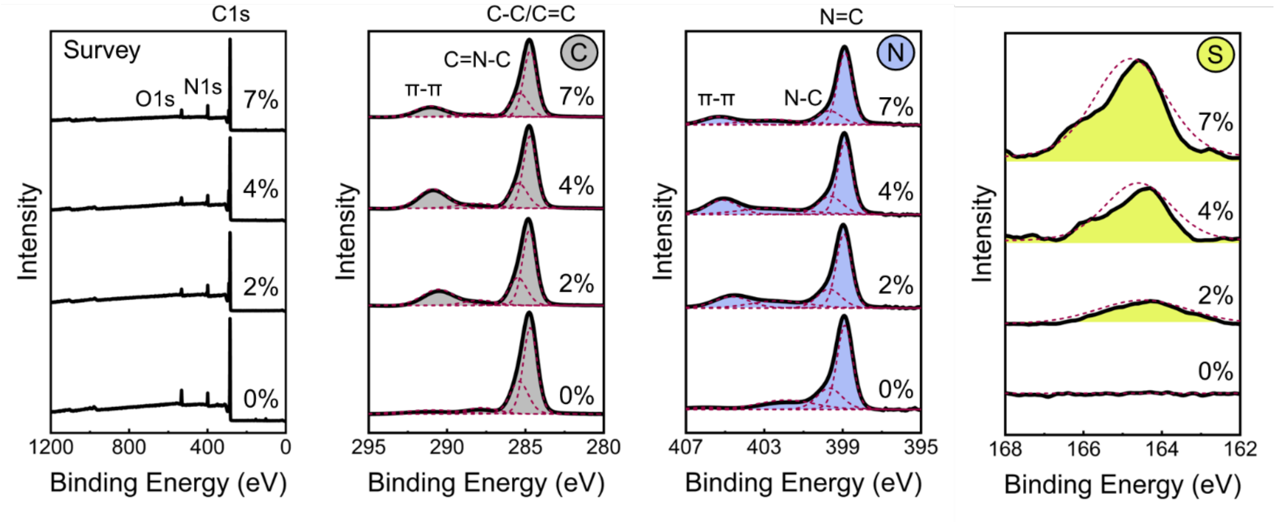

Figure S27. XPS scans of doped COF films used for construction of the band diagram.

All XPS scans were normalized to a C1s peak at 284.8eV to mitigate the effects of charging. Deconvoluted peak fits are shown as dashed red lines in the XPS.

	Raw XPS Peak Area					
COF Film	N=C	N-C	π-π	shoulder	S 2p	TDA %
1	2262	931	138	918	0	0
2	1988	749	707	705	26.7	2.1
3	1821	791	769	731	46.8	3.8
4	1840	580	438	530	86.8	7.4

Table S4. Raw deconvoluted XPS peak areas corresponding to the N1s and S2p spectra in **Figure S27**.

	Raw XPS Peak Position (N1s) [eV]			
TDA%	N=C	N-C	π-π	shoulder
0	398.9	399.7	405.9	401.9
2	398.9	399.7	404.7	402.2
4	398.9	399.6	405.1	402.5
7	398.9	399.7	405.4	402.6

Table S5. Raw deconvoluted XPS peak positions corresponding to the N1s spectra in **Figure S27** and peak areas in **Table S4**.

	Raw XPS Peak Area (C1s)			
TDA%	C-C/C=C	C=N-C	π-π	shoulder
0	17627	8787	3337	3075
2	14946	7314	7730	2501
4	14129	6620	8321	2764
7	12950	6184	4931	2104

Table S6. Raw deconvoluted XPS peak areas corresponding to the C1s spectra in **Figure S27**.

	Raw XPS Peak Position (C1s) [eV]			
TDA%	C-C/C=C	C=N-C	π-π	shoulder
0	284.7	285.4	290.9	287.8
2	284.7	285.5	290.5	287.9
4	284.7	285.4	290.9	288.1
7	284.7	285.4	291.1	288.0

Table S7. Raw deconvoluted XPS peak positions corresponding to the C1s spectra in **Figure S27** and peak areas in **Table S6**.

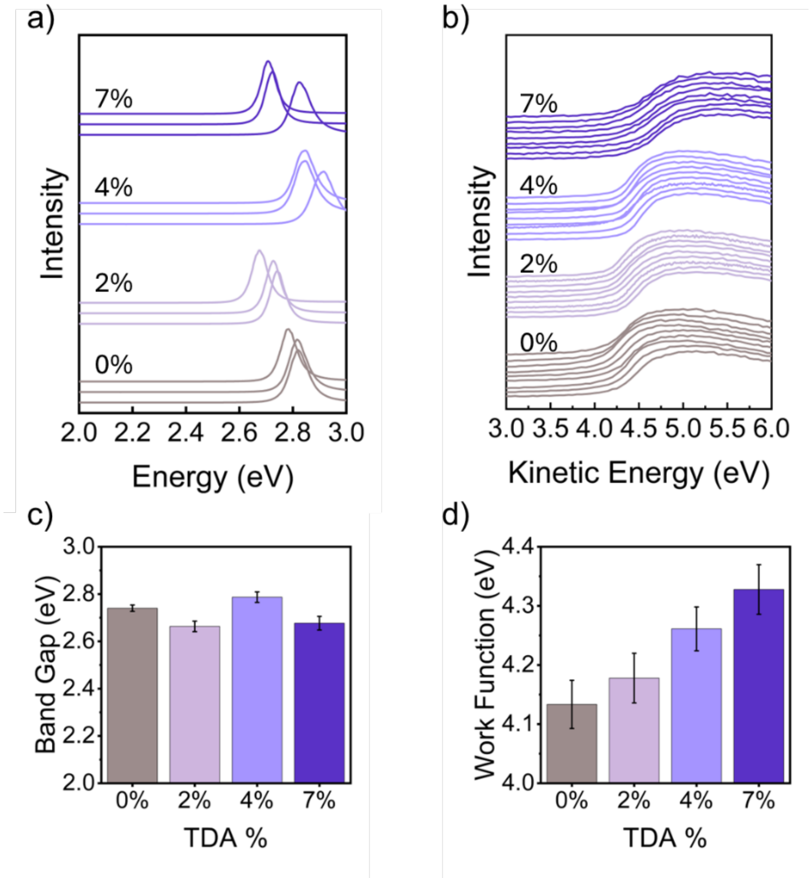

Figure S28. UV-Vis (a) and XPS (b) measurements of band gap and work function, respectively. Figures (c) and (d) present the average band gap and work function values calculated from the data in (a) and (b).

	Percentage of TDA Doping			
Energy (eV)	0%	2%	4%	7%
Band Gap:	2.74	2.66	2.79	2.68
Work Function:	4.13	4.18	4.26	4.33
VBM:	1.5	1.1	1.3	0.9

Table S8. Experimentally Determined Band Diagram Parameters

References

(1) Ren, X.; Sun, J.; Li, Y.; Bai, F. Primitive Functional Groups Directed Distinct Photocatalytic Performance of Imine-Linked Donor-Acceptor Covalent Organic Frameworks. *Nano Res.* **2024**, *17* (6), 4994–5001. https://doi.org/10.1007/s12274-024-6509-5.
(2) Rassie, C.; Olowu, R. A.; Waryo, T. T.; Wilson, L.; Williams, A.; Baker, P. G.; Iwuoha, E. I. Dendritic 7T-Polythiophene Electro-Catalytic Sensor System for the Determination of Polycyclic Aromatic Hydrocarbons. *International Journal of Electrochemical Science* **2011**, *6* (6), 1949–1967. https://doi.org/10.1016/S1452-3981(23)18158-2.
(3) Gedde, U. W. *Polymer Physics*; Springer Netherlands: Dordrecht, 1999. https://doi.org/10.1007/978-94-011-0543-9.
